# Physiological and transcriptional studies reveal Cr(VI) reduction mechanisms in the exoelectrogen *Cellulomonas fimi* Clb-11

**DOI:** 10.3389/fmicb.2023.1161303

**Published:** 2023-05-26

**Authors:** Lianbin Cao, Mingguo Lu, Mengrui Zhao, Yifan Zhang, Yiping Nong, Mengxue Hu, Ya Wang, Tongbiao Li, Fujia Chen, Mingcheng Wang, Junhe Liu, Enzhong Li, Hongmei Sun

**Affiliations:** School of Biological and Food Engineering, Huanghuai University, Zhumadian, Henan, China

**Keywords:** microbial fuel cells, Cr(VI) reduction, *Cellulomonas*, cell swelling, transcriptome

## Abstract

A facultative exoelectrogen, *Cellulomonas fimi* strain Clb-11, was isolated from polluted river water. This strain could generate electricity in microbial fuel cells (MFCs) with carboxymethyl cellulose (CMC) as the carbon source, and the maximum output power density was 12.17 ± 2.74 mW·m^−2^. In addition, Clb-11 could secrete extracellular chromate reductase or extracellular electron mediator to reduce Cr(VI) to Cr(III). When the Cr(VI) concentration was less than 0.5 mM in Luria-Bertani (LB) medium, Cr(VI) could be completely reduced by Clb-11. However, the Clb-11 cells swelled significantly in the presence of Cr(VI). We employed transcriptome sequencing analysis to identify genes involved in different Cr(VI) stress responses in Clb-11. The results indicate that 99 genes were continuously upregulated while 78 genes were continuously downregulated as the Cr(VI) concentration increased in the growth medium. These genes were mostly associated with DNA replication and repair, biosynthesis of secondary metabolites, ABC transporters, amino sugar and nucleotide sugar metabolism, and carbon metabolism. The swelling of Clb-11 cells might have been related to the upregulation of the genes *atoB*, *INO1*, *dhaM*, *dhal*, *dhak*, and *bccA*, which encode acetyl-CoA C-acetyltransferase, myo-inositol-1-phosphate synthase, phosphoenolpyruvate-glycerone phosphotransferase, and acetyl-CoA/propionyl-CoA carboxylase, respectively. Interestingly, the genes *cydA* and *cydB* related to electron transport were continuously downregulated as the Cr(VI) concentration increased. Our results provide clues to the molecular mechanism of Cr(VI) reduction by microorganisms in MFCs systems.

## 1. Introduction

Chromium is widely used in different industrial fields, such as pigment manufacturing, electroplating, mining, tanning, and welding (Poudel et al., 2021). It should be noted that the high mobility of Cr(VI) causes it to accumulate in aquatic biota and plants after entering sewage, and its strong biological toxicity poses a threat to ecology and human health (Xu et al., 2021). Therefore, the use of some effective remediation techniques to reduce the highly toxic Cr(VI) form to the less toxic Cr(III) form has become a hot topic of research (Wakeel et al., 2020).

Microbial fuel cells (MFCs) are promising environmentally friendly and renewable energy-producing devices that can convert biodegradable organic substrates to electricity by using electrochemically active microorganisms as anode biocatalysts. In particular, biocathode MFCs systems can simultaneously reduce Cr(VI) to Cr(III) in wastewater and produce electricity, and many studied have laid a foundation for research in this field (Zhang et al., 2022). Most exoelectrogens can be used as catalysts in the cathode chamber of biocathode MFCs to reduce Cr(VI) to Cr(III) by assisting the electron transfer between the electrode and Cr(VI) (Wu et al., 2018). To date, many studies have focused on improving the Cr(VI) reduction efficiency and electricity generation performance of MFCs (Ma et al., 2022). It was reported that medium solution (Xafenias et al., 2015), transition metal electrodes (Li et al., 2017), high-capacitance materials (Wang et al., 2018), and metal ions [such as Cu(II)] (Li and Zhou, 2019) can significantly improve the Cr(VI) reduction efficiency of biocathode MFCs. However, there are few reports on molecular biological techniques to regulate key gene expression in exoelectrogens to optimize the Cr(VI) reduction efficiency of MFCs, since the Cr(VI) reduction mechanism of exoelectrogens is still unclear.

The Cr(VI) reduction ability of microorganisms is genetically regulated in bacteria, and several genes have been reported to be responsible for Cr(VI) reduction. For example, the *chrB*, *chrA*, *chrC*, and *chrF* genes of *Ochrobactrum tritici*, *chrR* gene of *Pseudomonas aeruginosa*, *ruvB* gene of *Ochrobactrum tritici*, and *chr 2* gene of *Ralstonia metallidurans* have been reported to be associated with chromate resistance in these microorganisms (Thatoi et al., 2014). In addition to chromosomally encoded genes, bacterial plasmids contain resistance genes and encoded systems that protect bacterial cells from chromate-induced oxidative stress (Gadd, 2010). For example, the plasmid pMOL28 from *R. metallidurans* can encode the *chrA* chromate efflux pump to protect cells from chromate damage (Juhnke et al., 2002). A gene located on an *mre* operon in *Desulfovibrio desulfuricans* G20 can encode thioredoxin oxidoreductase, assisting in the reduction of Cr(VI) (Zou et al., 2013).

Microbial Cr(VI) reduction can be classified as intracellular or extracellular according to the site of occurrence. Soluble reductases represent one of the main modes of intracellular Cr(VI) reduction by microorganisms. These reductase proteins in bacteria that act as catalysts include NADH, NADPH, aldehyde oxidases, cytochrome p450, cytochrome c3, and Dt-diaphorase (Thatoi et al., 2014). In addition, several oxidoreductases with otherwise different metabolic functions can also catalyze Cr(VI) reduction, including nitroreductase, iron reductase, quinone reductases, hydrogenases, flavin reductases and NAD(P)H-dependent reductases (Thatoi et al., 2014). Gang et al. reported that the molybdate-related proteins ModA and ModC of *Shewanella oneidensis* MR-1 play important roles in removing chromate (Gang et al., 2019). Moreover, Cr(VI)-reducing enzymes can be exported into the medium and can reduce Cr(VI) in the extracellular space. However, these extracellular chromate reductases have not been isolated and identified and need to be further investigated in depth. Microbial resistance to Cr(VI) has been associated principally with a chromate transporter that pumps the metal ion out of the cell. In recent years, transcriptomic and proteomic analyses of the bacterial responses to chromium have indicated the existence of a more complex system related to the resistance and reduction of Cr(VI) (Lara et al., 2021). Therefore, it is scientifically important to elucidate the mechanism of Cr(VI) reduction in various microorganisms through transcriptomics.

In this work, we found that the exoelectrogen *Cellulomonas fimi* strain Clb-11 that we isolated secretes extracellular chromate reductase or extracellular electron mediator to reduce Cr(VI). Furthermore, we used whole genome sequencing and transcriptomic analysis to explore possible key Cr(VI) reduction genes of strain Clb-11. Eventually, several unreported genes involved in chromate reduction and transport were screened. This study provides a novel reference for explaining the Cr(VI) reduction mechanism of exoelectrogens.

## 2. Materials and methods

### 2.1. Isolation and identification of Clb-11

Twenty milliliters of river water was used as the anode inoculum in the MFCs. When the output voltage of the MFCs peaked, the exoelectrogenic species was thought to have been enriched in the anode chamber. Then, the anolyte was removed and streaked on LB medium plates. Single colonies were picked and restreaked more than five times until the colonies of identical morphology and shape were obtained. Finally, the pure culture was named strain Clb-11. The single clones were used as templates for PCR analysis with the universal primers 27F (5′-AGA GTT TGA TCA TGG CTC AG-3′) and 1492R (5′-TAC GGC TAC CTT GTT ACG ACT T-3′), and the 16S rDNA sequences were compared against the NCBI database. The whole genome was sequenced by the biotechnology company Novogene.

### 2.2. Genome sequencing of Clb-11

Whole-genome sequencing of Clb-11 was completed by Novogene. Genomic DNA was extracted with the SDS method. The harvested DNA was detected by agarose gel electrophoresis and quantified by a Qubit^®^ 2.0 Fluorometer (Thermo Scientific). The library was constructed using the Nanopore PromethION platform and Illumina NovaSeq platform. GeneMarkS (Version 4.17) software was used for coding gene prediction of newly sequenced genomes. IslandPath-DIOMB software (Version 0.2) was used to predict gene islands. The whole genome map of the sample was displayed using Circos software.

### 2.3. Analysis and determination of chromium levels

After cells were cultured in growth medium containing Cr(VI) at various concentrations, the residual Cr(VI) was analyzed using the colorimetric standard method (Zhang et al., 2015). A linear relationship (*R*^2^ = 0.9999) was observed between the Cr(VI) concentrations and solution absorbances at 540 nm with Cr(VI) concentrations of 0–20 μg·mL^−1^.

### 2.4. Extracellular chromate reductase or extracellular electron mediator assay

Clb-11 was cultured in LB medium for 72 h. Then, the sterile supernatant was obtained by centrifugation (80.00 g, 15 min and 4°C) and filtration (0.22 μm filter membrane). The sterile supernatant was used as the crude extract of extracellular chromate reductase. Approximately 0.1 mM Cr(VI) was added to the sterile supernatant, and the change in Cr(VI) concentration was observed after 3 h. LB medium without inoculated Clb-11 was used as the control.

### 2.5. Morphological analysis

After being cultured in LB medium with different Cr(VI) concentrations (0, 0.1, 0.3, 0.5, and 0.7 mM) for 72 h, the Clb-11 cells were centrifuged (1,500 × g, 20 min and 4°C) to obtain the cell pellets. Then, morphological analysis of the Clb-11 cells was performed on a Hitachi S-3400 N scanning electron microscope (Hitachi, Tokyo, Japan).

### 2.6. Transcriptomic analysis

After being cultured in LB medium with different Cr(VI) concentrations (0, 0.2, and 0.5 mM) until the OD_600 nm_ reached 0.6–0.8, the Clb-11 cells were centrifuged (1,500 × g, 20 min and 4°C) to obtain the cell pellets. Then, the samples were sent to Novogene for transcriptomic analysis.

RNA integrity was assessed using the RNA Nano 6,000 Assay Kit for the Bioanalyzer 2,100 system (Agilent Technologies, CA, United States). The clustering of the index-coded samples was performed on a cBot Cluster Generation System using TruSeq PE Cluster Kit v3-cBot-HS (Illumina). Rockhopper was used to identify novel genes, operons, TSSs, TTSs and cis-natural antisense transcripts. HTSeq v0.6.1 was used to count the read numbers mapped to each gene. The fragments per kilobase of exon model per million mapped fragments (FPKM) value of each gene was calculated based on the length of the gene and read count mapped to the gene. Differential expression analysis of two conditions/groups (two biological replicates per condition) was performed using the DESeq R package (1.18.0). Gene Ontology (GO) enrichment analysis of differentially expressed genes was implemented by the GOseq R package. We used KOBAS software to test the statistical enrichment of differentially expressed genes in KEGG pathways.

### 2.7. MFCs construction and operating conditions

In the two-chamber MFCs, the chambers each had a working volume of 120 mL, contained a magnetic stir bar and were operated anaerobically. The anode chamber was filled with growth medium, which was similar to that previously reported (Cao et al., 2020), containing strain Clb-11 with CMC as the carbon source. The method of strain Clb-11 inoculation into the MFCs anode was is similar to that previously reported (Cao et al., 2020). A carbon rod (geometric surface area of 6.48 cm^2^) connected by a titanium wire was used as the anode. Meanwhile, carbon felt (30 mm × 20 mm × 5 mm) connected by titanium wires was used in the cathode chamber, which contained 50 mM potassium ferricyanide solution in 100 mM phosphate buffer (pH 7.0). The output voltages of the MFCs were recorded using a Keithley instrument (model 2400) with 1,000 Ω resistance connected in the circuit. All MFCs were operated at 30 ± 1°C.

### 2.8. Statistical analysis

The data were analyzed by SPSS version 24.0 for Windows (SPSS Inc., Chicago, IL, United States). Continuous variables with a normal distribution are presented as the mean ± standard deviation (SD). Means of two continuous normally distributed variables were compared by independent samples Student’s t test. A value of *p* < 0.05 was considered significant.

## 3. Results and discussion

### 3.1. Isolation and draft genome sequencing of Clb-11

Clb-11 was isolated from polluted river water collected in Henan Province of China. Comparison with sequences in GenBank suggested that Clb-11 belongs to the genus *Cellulomonas* and is related to TMV7-A2 (with a similarity of 99.64%) (Supplementary Figure S1). The genome of Clb-11 was sequenced by Novogene. The results showed that the estimated genome size was 4,486,195 base pairs (bp), with an N_50_ read length of 12,302 bp and a GC content of 74.41% (Supplementary Tables S1, S2). GeneMarkS (Version 4.17) software was used to predict the coding genes in the Clb-11 genome, and 4,052 protein-coding genes were predicted. tRNAscan-SE (Version 1.3.1), rRNAmmer (Version 1.2), and cmsearch (Version 1.1rc4) predicted 45, 6, and 0 tRNA-, rRNA-, and sRNA-coding genes, respectively. Genomic islands (GIs) are genomic regions in some bacteria, phages or plasmids that have been integrated into the microbial genome by horizontal transfer of genes. GIs can be associated with a variety of biological functions, such as pathogenic mechanisms and organismal adaptations. IslandPath-DIOMB (Version 0.2) predicted 9 GIs, and the gene distribution statistical map is shown in Supplementary Figure S2.

**Figure 1:**
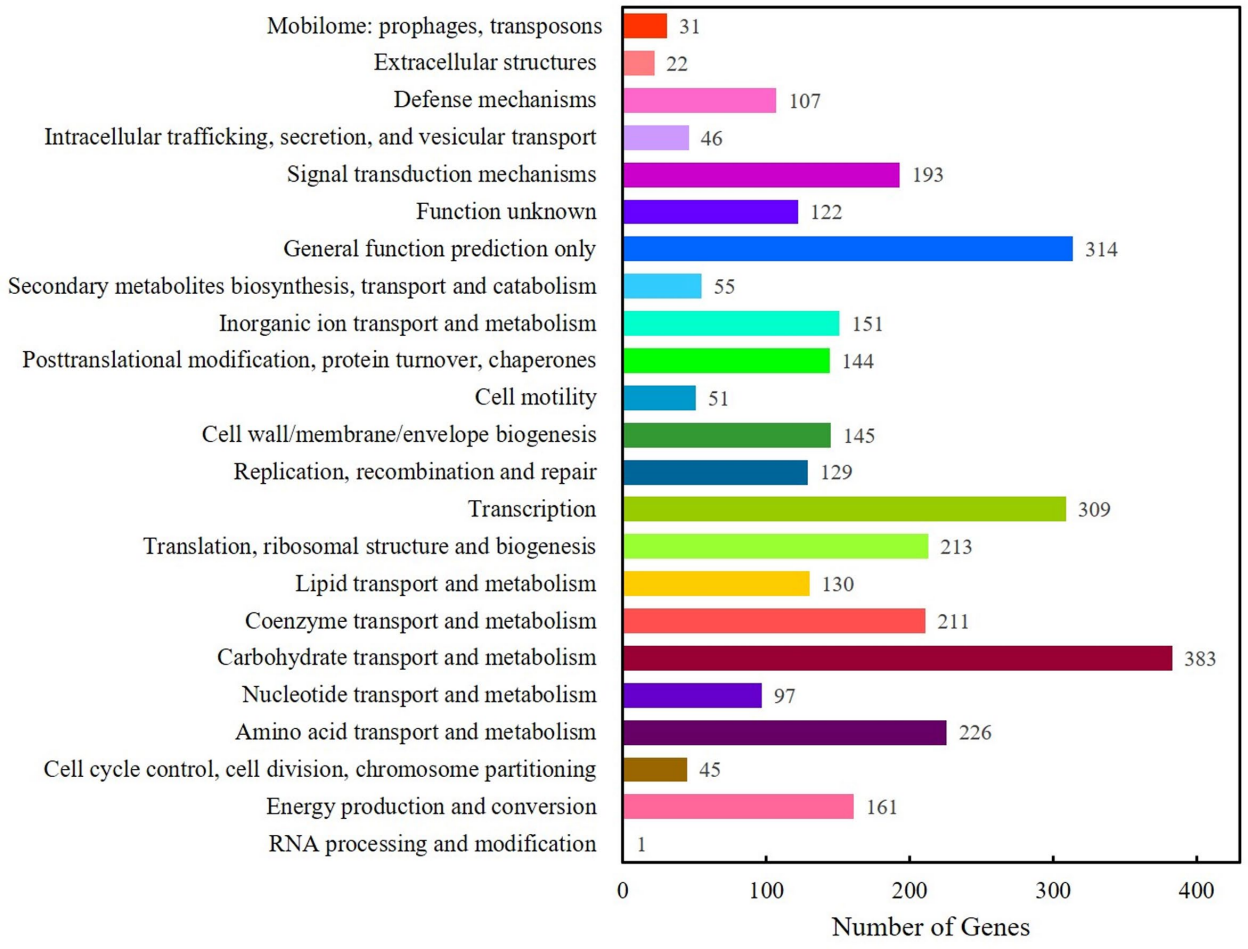
Gene function annotation of Clb-11 by Cluster of Orthologous Groups of proteins (COG).

IPRscan software was used to annotate the gene functions of Clb-11 through the Gene Ontology database, and the results are shown in Supplementary Figure S3. The terms in the molecular function category were mainly related to transporter activity, structural molecule activity, receptor regulator activity, and protein binding transcription factor activity. The terms in the cellular component category were mainly related to virion part, virion, organelle part, and organelle. The terms in the biological process category were mainly related to viral reproduction, signaling, response to stimulus, reproductive process, and reproduction. A total of 4,052 genes were annotated (Supplementary Figure S4) through the Kyoto Encyclopedia of Genes and Genomes (KEGG) database. Based on functional annotation, the KEGG database provided metabolic pathways that were divided into six categories. There were 129, 200, 194, 58, 1,553, and 49 genes involved in cellular processes, environmental information processing, genetic information processing, human diseases, metabolism, and organismal systems, respectively. The Cluster of Orthologous Groups of proteins (COG) annotation of the Clb-11 genome is shown in Figure 1; a total of 4,052 genes were annotated. Most of the genes were assigned to functions associated with carbohydrate transport and metabolism, transcription, amino acid transport and metabolism, translation, ribosomal structure and biogenesis, and coenzyme transport and metabolism. However, genes of unknown function also exhibited a high abundance. Gene function annotation of Clb-11 was performed by the Transporter Classification Database (TCDB), and the results are shown in Supplementary Figure S5. Most of the genes were assigned to functions of primary active transporters. In addition, SignalP (Version 4.1) and TMHMM (Version 2.0c) were used to predict secreted proteins, and Clb-11 was found to encode 102 secreted proteins (Supplementary Table S4). These results indicated that Clb-11 had strong capacities for substance transport and stress tolerance. These characteristics provided the basis for the Cr(VI) reduction capacity and electricity generation capacity of Clb-11. The final complete whole-genome map of Clb-11 with 0 gaps is shown in Supplementary Figure S6.

### 3.2. Cr(VI) reduction ability of Clb-11

**Figure 2:**
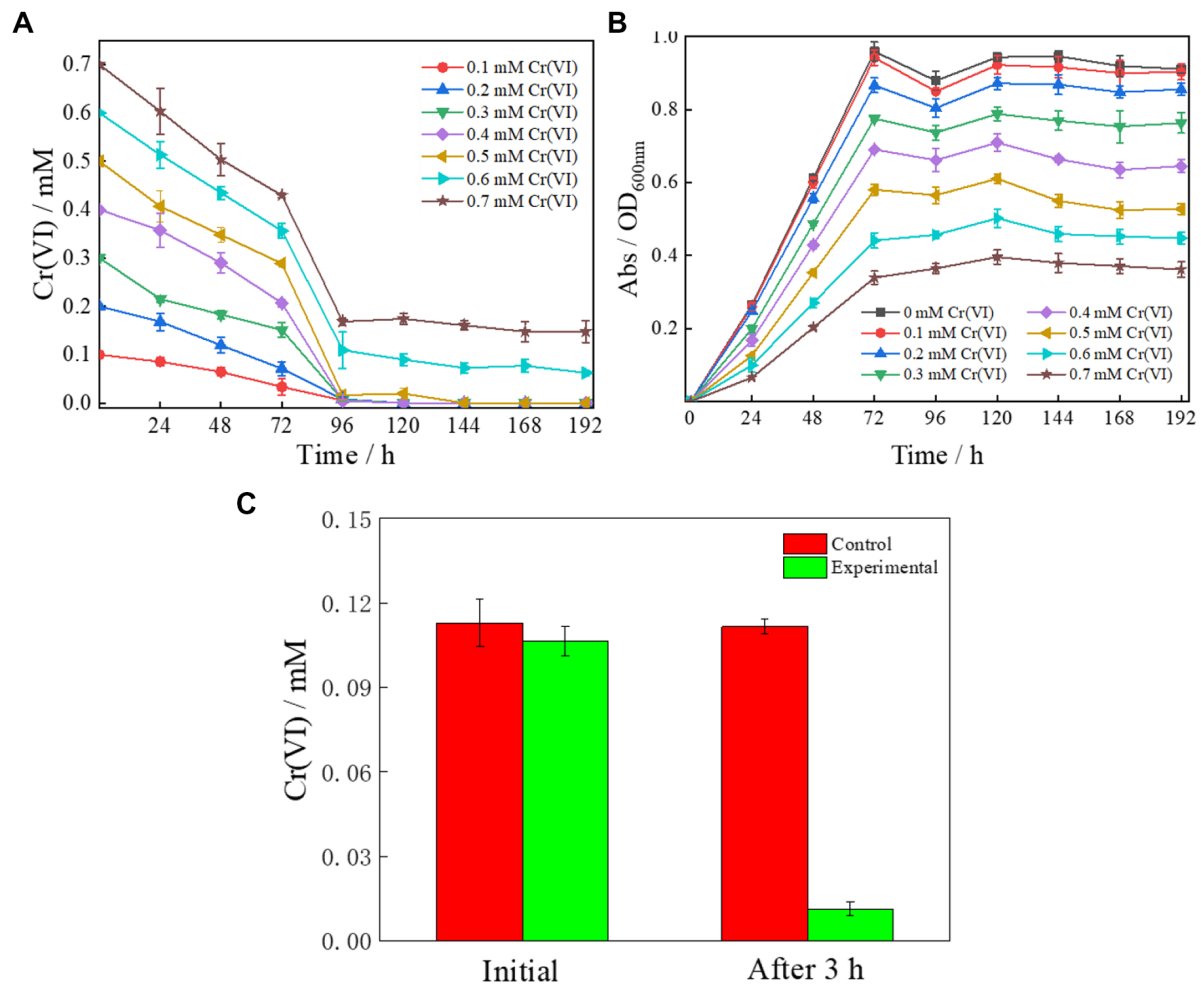
Cr(VI) reduction performance **(A,B)** and extracellular chromate reductase secretion capacity **(C)** of Clb-11. **(A)** Dependence of Cr(VI) concentrations on time with Clb-11 in LB medium with different initial concentrations of Cr(VI). **(B)** Growth curves of Clb-11 in LB medium with different initial concentrations of Cr(VI). **(C)** Clb-11 was cultured in LB medium for 72 h, centrifuged and filtered to obtain sterile supernatant. Then, Cr(VI) was added, and the change in Cr(VI) concentration was observed after 3 h. LB medium without inoculated Clb-11 was used as the control.

Some species of *Cellulomonas* have the ability to reduce Cr(VI) (Xu et al., 2005). As expected, Clb-11 was found to have a similar ability to reduce Cr(VI). Therefore, Clb-11 was inoculated into LB medium containing 0.1–0.7 mM Cr(VI) (Figures 2A,B). Gradual reduction of Cr(VI) by Clb-11 was observed in the LB medium (Figure 2A). At a concentration of 0.5 mM, Cr(VI) was completely reduced within 144 h (Figure 2A). When the Cr(VI) concentration was increased to 0.6 and 0.7 mM, 0.062 ± 0.0053 mM and 0.15 ± 0.022 mM Cr(VI) remained in the LB medium after 192 h, respectively (Figure 2A). Under different Cr(VI) concentrations, the growth of Clb-11 in LB medium varied. As the Cr(VI) concentration increased, the cell density of Clb-11 decreased gradually (Figure 2B). However, with Cr(VI) concentrations from 0 to 0.7 mM, the OD_600 nm_ reached maximum values of 0.96 ± 0.026, 0.94 ± 0.021, 0.87 ± 0.02, 0.78 ± 0.0056, 0.69 ± 0.0050, 0.58 ± 0.015, 0.44 ± 0.021, and 0.34 ± 0.018 at 72 h (Figure 2B). These results illustrated that Cr(VI) at excessive concentrations can inhibit the microbial reduction of Cr(VI), and Cr(VI) can be harmful for cell growth. Cr(VI) has toxic side effects on microorganisms and can inhibit cell growth (Cao et al., 2020). Furthermore, Cr(VI) at excessive concentrations can inhibit hexavalent chromium reductase activity (Hu et al., 2018). The reduction ratio of Cr(VI) was the highest within 72–96 h at Cr(VI) concentrations from 0.2 to 0.7 mM (Figure 2A and Supplementary Table S5). This may be because the cell density peaked at 72 h (Figure 2B), corresponding to the peak reduction ratio. In addition, we studied the extracellular secretion of Clb-11. It was found that extracellular secretion of Clb-11 reduced Cr(VI) from 0.11 ± 0.0052 mM to 0.011 ± 0.0026 mM within 3 h (Figure 2C). These results showed that Clb-11 can secrete extracellular chromate reductase or extracellular electron mediator to reduce Cr(VI). However, the Cr(VI) reduction mechanism of extracellular chromate reductase or extracellular electron mediator has not been reported. Therefore, transcriptomic research on Clb-11 may be the key to solving this problem.

**Figure 3:**
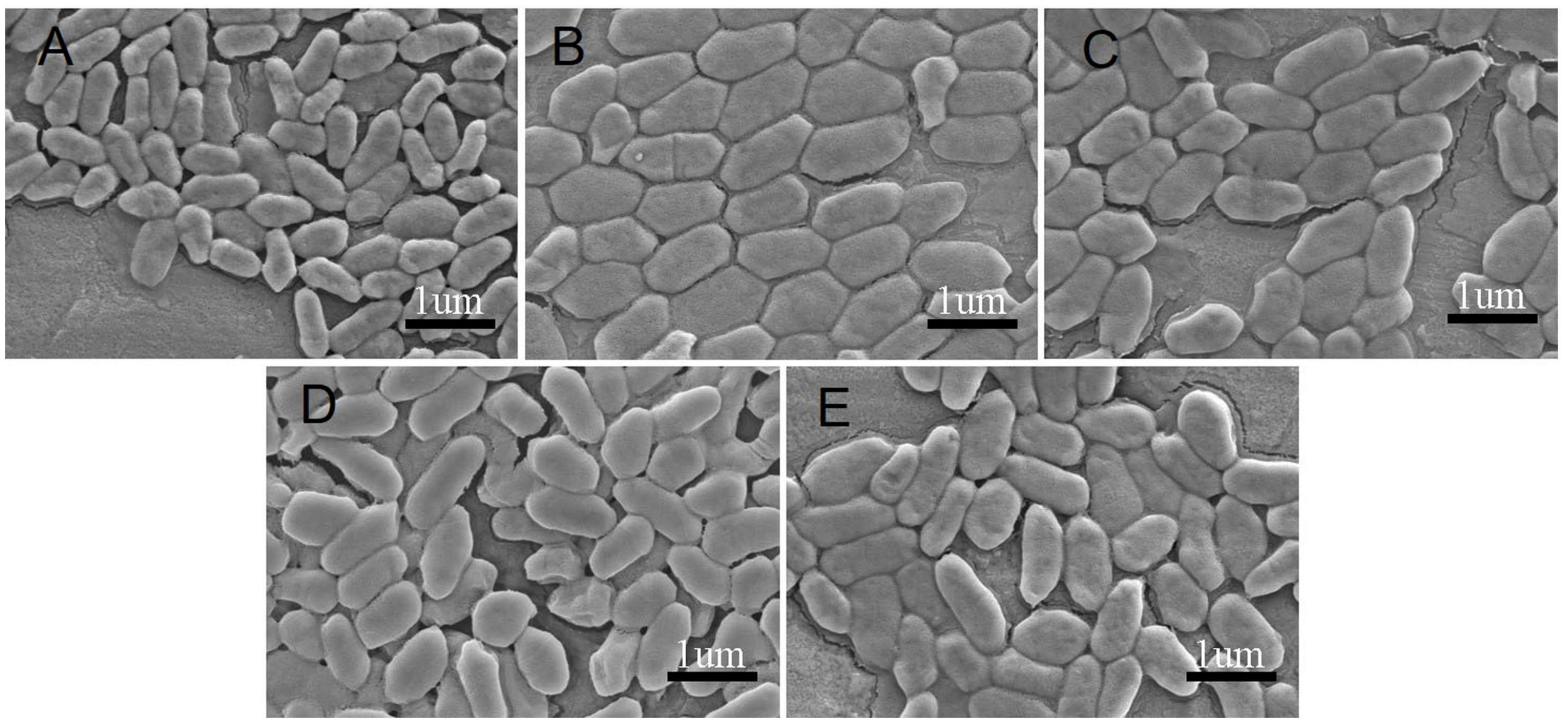
SEM of Clb-11 cells grown in LB medium for 192 h with different Cr(VI) concentrations: 0 mM **(A)**, 0.1 mM **(B)**, 0.3 mM **(C)**, 0.5 mM **(D)**, and 0.7 mM **(E)**.

The morphological characteristics of Clb-11 grown for 192 h with different Cr(VI) concentrations (0, 0.1, 0.3, 0.5, and 0.7 mM) were studied using scanning electron microscopy (SEM) (Figure 3). Clb-11 is an irregular bacillus (Figure 3A). Without Cr(VI) in the LB medium, the cell length of Clb-11 ranged from 0.4 to 0.7 μm (Figure 3A). With a Cr(VI) concentration of 0.1 mM, the cell length of Clb-11 ranged from 0.7 to 1.2 μm (Figure 3B). When grown with Cr(VI), cells swell as a self-protection mechanism (Chourey et al., 2006). However, the cell length of Clb-11 did not change as the Cr(VI) concentration increased (Figures 3C–E). The results indicated that the Clb-11 cells swelled significantly in the presence of Cr(VI). Similar results were reported for *Pseudomonas* (Dogan et al., 2011).

### 3.3. Transcriptional profile of Clb-11 cells adapted to Cr(VI)

To study the Cr(VI) reduction mechanism of Clb-11, the differential transcriptional profile of Clb-11 cells preadapted to 0.2 and 0.5 mM Cr(VI) relative to the control in LB medium without Cr(VI) was evaluated by RNA-Seq. Three replicate experiments under each condition were conducted from the mid-exponential growth phase (OD_600 nm_ = 0.6) for RNA extraction, library construction, and sequencing. The Pearson correlation (*R*^2^ > 0.92) (Patro et al., 2013) between the Clb-11 cell samples showed that the replicate experiments had good correlation (Supplementary Figure S7). Principal component analysis (PCA) of Clb-11 cell samples also showed that the intergroup samples were dispersed and that the intragroup samples were clustered together (Supplementary Figure S8). These results indicated that the sample selection was reasonable and that the results of transcriptome analysis were reliable.

**Figure 4:**
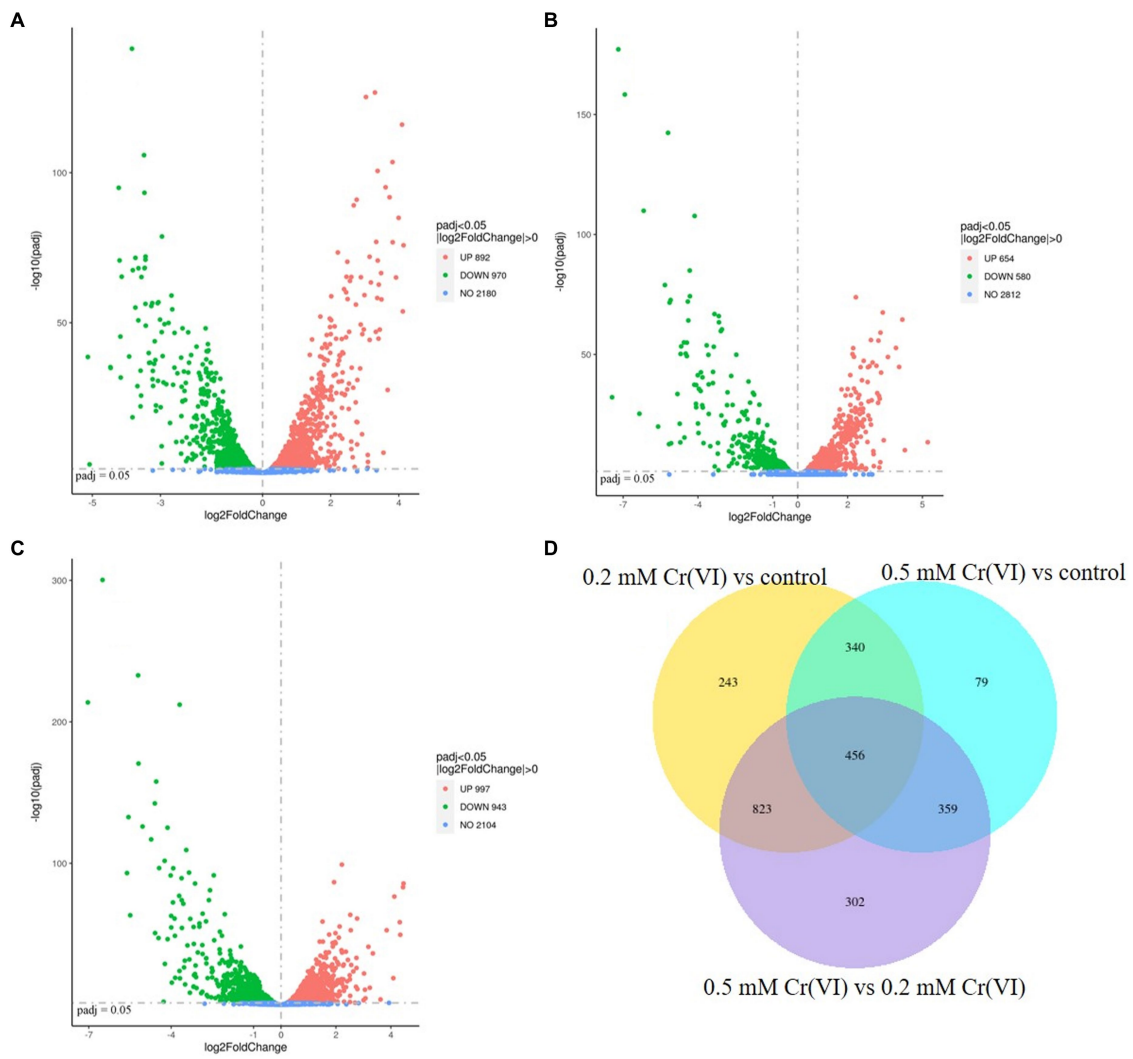
Volcano plot displaying differentially transcribed genes between **(A)** 0.2 mM Cr(VI)-adapted cells and controls, **(B)** 0.5 mM Cr(VI)-adapted cells and controls, and **(C)** 0.5 mM Cr(VI)-adapted cells and 0.2 mM Cr(VI)-adapted cells. Each dot represents an open reading frame (ORF). ORFs with padj <0.05 and |Log2FoldChange| > 0 were considered differentially transcribed. Upregulated genes are indicated by red dots, and downregulated genes are indicated by green dots. **(D)** Venn diagram of differentially transcribed genes.

The RNA-Seq results showed that there were differences between the Cr(VI)-adapted cells and controls (Figure 4). Moreover, there were differences between the Cr(VI)-adapted cells at 0.5 and 0.2 mM Cr(VI) (Figure 4). Using padj <0.05 and |Log2FoldChange| > 0 as cutoffs, compared with the controls, 970 genes were downregulated, while 892 were upregulated, in the presence of 0.2 mM Cr(VI) (Figure 4A). Using a padj <0.05 and |Log2FoldChange| > 0 cutoff, compared with the controls, 580 genes were downregulated, while 654 were upregulated, in the presence of 0.5 mM Cr(VI) (Figure 4B). In addition, using padj <0.05 and |Log2FoldChange| > 0 as cutoffs, 943 genes were downregulated in the presence of 0.5 mM Cr(VI) compared with 0.2 mM Cr(VI), while 997 were upregulated in the presence of 0.5 mM Cr(VI) (Figure 4C). These data suggested that the transcription level in Clb-11 cells changed when Cr(VI) was present in the medium, which is consistent with previous reports on *Klebsiella* sp. (Lara et al., 2021). The increase in the concentration of Cr(VI) further affected the transcription level in Clb-11 cells. The same result was observed in the differential gene cluster heatmap (Supplementary Figure S9). When Clb-11 cells grow in the presence of Cr(VI), the transcriptional regulation system for genes changes as a self-protection mechanism (Chourey et al., 2006).

**Figure 5:**
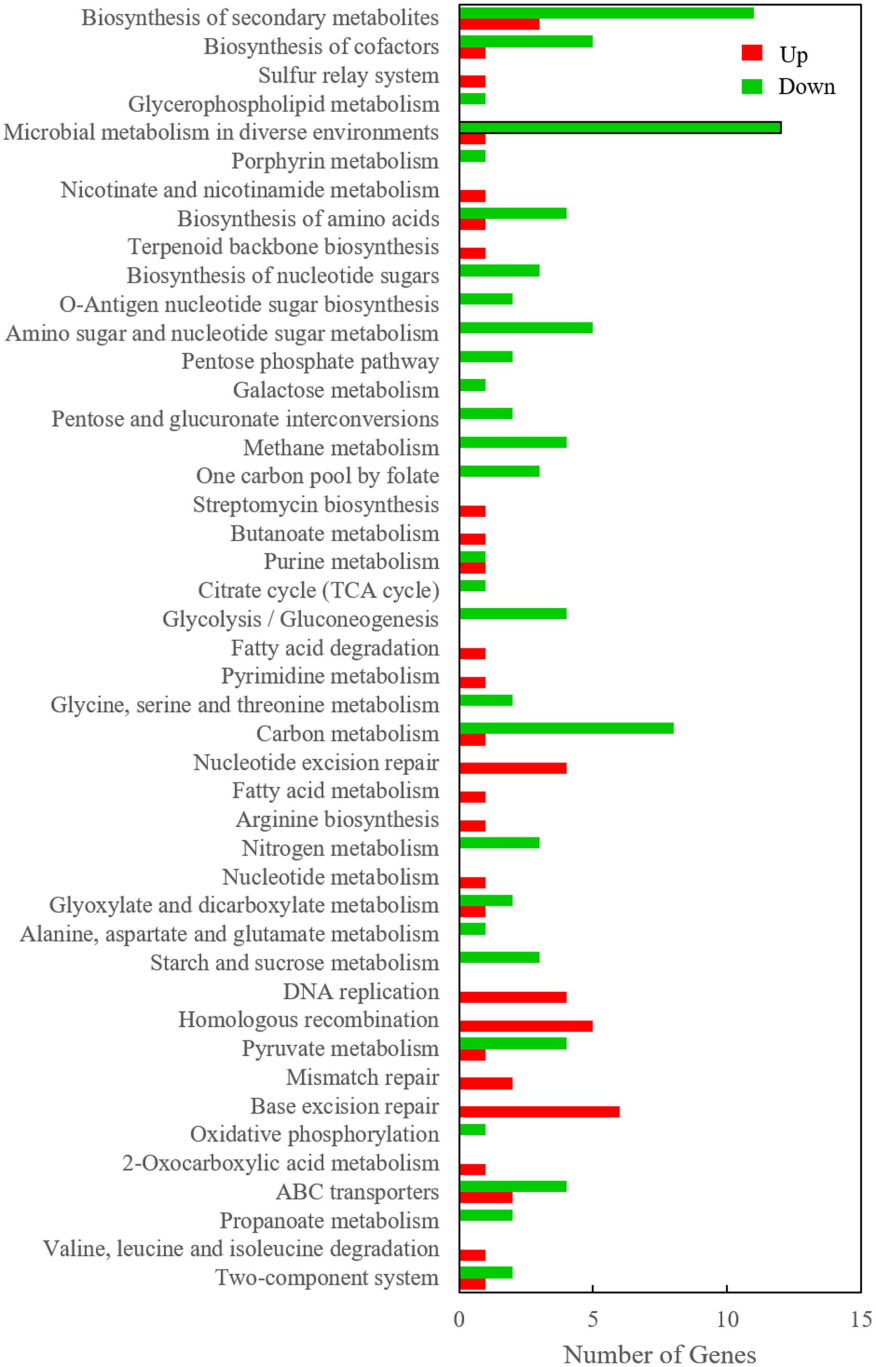
KEGG pathway enrichment of 99 upregulated genes and 78 downregulated genes in the Clb-11 genome.

However, it is very difficult to identify the key genes related to Cr(VI) reduction from among the large number of differentially expressed genes, so subsequent verification is extremely important. In this study, a Venn diagram of differentially transcribed genes between 0.2 mM Cr(VI)-adapted cells and controls, 0.5 mM Cr(VI)-adapted cells and controls, and 0.5 mM Cr(VI)-adapted cells and 0.2 mM Cr(VI)-adapted cells was constructed (Figure 4D). A total of 456 overlapping differentially expressed genes, which were closely related to the Cr(VI) reduction mechanism of Clb-11, were identified among the three groups (Figure 4D). Among the 456 differentially expressed genes, the expression levels of 99 genes were continuously upregulated with increasing Cr(VI) concentration (Supplementary Table S6), the expression levels of 78 genes were continuously downregulated with increasing Cr(VI) concentration (Supplementary Table S7), the expression levels of 98 genes were first upregulated and then downregulated with increasing Cr(VI) concentration, and the expression levels of 181 genes were first downregulated and then upregulated with increasing Cr(VI) concentration. As the Cr(VI) concentration in the medium increased, the cells responded accordingly to resist the toxic effect of Cr(VI). The 99 upregulated genes may play a key role in cellular resistance to Cr(VI) toxicity. The 78 downregulated genes may be associated with the damage caused to cells by Cr(VI) toxicity. Therefore, KEGG pathway enrichment analysis of 99 upregulated genes (Supplementary Table S6) and 78 downregulated genes (Supplementary Table S7) in Clb-11 cells was performed (Figure 5). The 99 upregulated genes were mostly related to nucleotide excision repair, DNA replication, homologous recombination, base excision repair, and biosynthesis of secondary metabolites, and the 78 downregulated genes were mostly related to carbon metabolism, pyruvate metabolism, glycolysis/gluconeogenesis, methane metabolism, amino sugar and nucleotide sugar metabolism, biosynthesis of amino acids, microbial metabolism in diverse environments, biosynthesis of cofactors, ABC transporters, and biosynthesis of secondary metabolites (Figure 5 and Supplementary Tables S6, S7). The KEGG pathway enrichment analysis results showed that these upregulated genes may be related to the Cr(VI) resistance of cells, and the downregulated genes may be related to the cytotoxicity of Cr(VI). However, most of the upregulated and downregulated genes could not be annotated in KEGG pathways (Supplementary Tables S6, S7).

### 3.4. DNA repair and replication

Cr(VI) elicits DNA damage both directly and by inducing cellular oxidative stress (Meaza et al., 2020). As the Cr(VI) concentration increased in the medium, the *ruvB* (Clb.11_GM001926), *recA* (Clb.11_GM002452), *dnaB* (Clb.11_GM004012), *uvrA* (Clb.11_GM002131), *nrdA* (Clb.11_GM000387), and *uvrD* (Clb.11_GM001182) genes, which are related to DNA repair and replication, were continuously upregulated (Supplementary Table S6), which was similar to the result of a previous report (Hu et al., 2005; Chourey et al., 2006; Zhang et al., 2014; Lara et al., 2021). However, the genes *nei* (Clb.11_GM002457) encoding endonuclease VIII, *mutM* (Clb.11_GM002494) encoding formamidopyrimidine-DNA glycosylase, *xthA* (Clb.11_GM000352/Clb.11_GM001218) encoding exodeoxyribonuclease III, *ligA* (Clb.11_GM002940) encoding the NAD-dependent DNA ligase LigA, *polA* (Clb.11_GM002149) encoding DNA polymerase I, *holA* (Clb.11_GM001579) encoding DNA polymerase III subunit delta, *ruvA* (Clb.11_GM001925) encoding the Holliday junction DNA helicase RuvA, *uvrC* (Clb.11_GM002128) encoding excinuclease ABC subunit C, *imuB* (Clb.11_GM002278) encoding a Y-family DNA polymerase, *dinB* (Clb.11_GM002813) encoding DNA polymerase IV, *dcm* (Clb.11_GM000067) encoding DNA (cytosine-5)-methyltransferase 1, *radA* (Clb.11_GM000379) encoding DNA repair protein RadA/Sms, *dnaE2* (Clb.11_GM002277) encoding an error-prone DNA polymerase, *sbcD* (Clb.11_GM000184) and *sbcC* (Clb.11_GM000185) encoding the DNA repair protein SbcD/Mre11 and SbcC/Rad50, *lhr* (Clb.11_GM002456) encoding the ATP-dependent helicase Lhr and Lhr-like helicase, and *ftsK* (Clb.11_GM001380) encoding the DNA segregation ATPase FtsK/SpoIIIE were upregulated, which has not previously been reported (Supplementary Table S6). These genes were also involved in DNA repair and replication, and their upregulation indicated that Cr(VI) may damage DNA replication in Clb-11 and that the damage increases as the Cr(VI) concentration increases.

### 3.5. Transporters and cytochromes

Bacteria exposed to excessive levels of metals generally express genes encoding transporters (Zhang et al., 2014). ABC transporters play an important role in bacterial resistance. As the Cr(VI) concentration increased in the medium, the genes *ganP* (Clb.11_GM001640), encoding am arabinogalactan oligomer/maltooligosaccharide transport system permease protein, and *ganO* (Clb.11_GM001639), encoding an arabinogalactan oligomer/maltooligosaccharide transport system substrate-binding protein of ABC transporters, were continuously upregulated (Supplementary Table S6).

Persistent upregulation was also detected for four putative ABC transporter protein-encoding genes (Supplementary Table S6). Arabinogalactan oligomers enhance microbial tolerance (Taguchi et al., 2004), and maltooligosaccharides play a positive role in microbial proliferation and resistance (Soto, 2013). Therefore, arabinogalactan oligomers and maltooligosaccharides may be heavily transported by Clb-11 cells to resist Cr(VI) damage. In addition, two MFS transporter-encoding genes, *lmrB* (Clb.11_GM003261) and *mef* (Clb.11_GM003910), which are related to cellular resistance, were upregulated (Supplementary Table S6). However, as the Cr(VI) concentration increased in the medium, no upregulation of genes associated with chromate transporters was found. The same result was reported in *S. oneidensis* MR-1 (Bencheikh-Latmani et al., 2005) and *Klebsiella* sp. strain AqSCr (Lara et al., 2021). Moreover, the transcript level of the known chromate transporter encoding gene *chrA* (Clb.11_GM003029) was low in Clb-11 cells grown with 0.5 mM Cr(VI) (Supplementary Table S8). These data illustrated that microorganisms preferentially increase their resistance in chromium-containing environments to protect themselves. Some chromate transporter-encoding genes in Clb-11 cells were unannotated. In addition, it was reported that *Cellulomonas* species could reduce Cr(VI) *via* hexavalent chromium reductase *in vitro* (Cao et al., 2020), which might be why the chromate transporter-encoding gene was not upregulated.

When cells were exposed to chromium, the levels of some proteins were downregulated. As the Cr(VI) concentration increased in the medium, the genes *cydC* and *cydD* encoding ABC-type transporters, *msmE* encoding a raffinose/stachyose/melibiose transport system substrate-binding protein, and *msmF* encoding a raffinose/stachyose/melibiose transport system permease protein of ABC transporters were continuously downregulated (Supplementary Table S7). Furthermore, unexpectedly, the cytochrome-encoding genes, which were related to Cr(VI) reduction, were not upregulated in the Clb-11 cells grown with 0.2 mM Cr(VI). In contrast, the genes *qcrB* (Clb.11_GM001787) encoding ubiquinol-cytochrome *c* reductase cytochrome *b* subunit, *cydA* (Clb.11_GM001485) encoding cytochrome *bd* ubiquinol oxidase subunit I, *cydB* (Clb.11_GM001484) encoding cytochrome *bd* ubiquinol oxidase subunit II, *ccdA* (Clb.11_GM001767/Clb.11_GM000996) encoding cytochrome *c*-type biogenesis protein, and *ctaA* (Clb.11_GM002325) encoding cytochrome *c* oxidase assembly protein subunit 15 were downregulated in the Clb-11 cells grown with 0.2 mM Cr(VI) (Supplementary Table S9). When the concentration of Cr(VI) was increased to 0.5 mM, the cytochrome-encoding genes *cydA* and *cydB* were continuously downregulated (Supplementary Table S9). The downregulation of *cydA-* and *cydB*-encoding genes probably reduced the energy supply available for RNA and protein synthesis (Strauss et al., 2004), thereby restoring the balance in the synthesis of different cellular components to resist the damage caused by Cr(VI). In addition, the downregulation of the *cydA* and *cydB* genes led to a decrease in the expression of cytochrome *bd*. The *cydD* and *cydC* genes, encoding an ABC-type transporter, are required for the assembly of cytochrome *bd* (Cruz-Ramos et al., 2004). The expression of the *cydD* and *cydC* genes continued to decrease, possibly due to the inhibition of metabolic accumulation. However, the reason for the downregulation of the *msmE* and *msmF* genes requires further study. These data showed that cytochrome *bd* is not a factor influencing the microbial reduction of Cr(VI). The downregulation of the genes *qcrB*, *ccdA* and *ctaA* indicated that when the Cr(VI) concentration was low, cytochrome *c* did not play a decisive role in the removal of Cr(VI) from the cells. Chromate reductase may play a key role in this process, and *Cellulomonas* species can secrete chromate reductase to reduce Cr(VI) extracellularly (Cao et al., 2020). However, the known chromate reductase-encoding gene *chrR* (Clb.11_GM003425) was not upregulated, and no other chromate reductase-encoding gene was found in Clb-11 (Supplementary Table S8). Therefore, other chromate reductase-encoding genes in Clb-11 cells need to be identified in the future. However, cytochrome *bd* is essential for electron transport in exoelectrogens (Borisov et al., 2011), and this might be the reason for the electricity production of *Cellulomonas* being reduced when Cr(VI) was present in the anode medium (Cao et al., 2020).

In addition, when the concentration of Cr(VI) was increased to 0.5 mM, the cytochrome-encoding genes *qcrB* (Clb.11_GM001787), *ccdA* (Clb.11_GM001767), *qcrA* (Clb.11_GM001788) encoding ubiquinol-cytochrome *c* reductase iron–sulfur subunit, *qcrC* (Clb.11_GM001789) encoding ubiquinol-cytochrome *c* reductase cytochrome *c* subunit, and ctaD/E/C (Clb.11_GM001766/Clb.11_GM001790/Clb.11_GM001765) encoding cytochrome *c* oxidase subunit I/III/II were upregulated compared to the expression level under 0.2 mM Cr(VI) (Supplementary Table S9). These data showed that when the Cr(VI) concentration was high in the medium, the cells began to express a large amount of cytochrome *c* and auxiliary proteins to reduce Cr(VI).

### 3.6. Cell envelope

The Clb-11 cells swelled significantly in the presence of Cr(VI) (Figure 3). The swelling of Clb-11 cells caused the surface area of the cell membrane to increase. Therefore, the genes related to biofilm synthesis were analyzed. The results indicate that the genes *atoB* (Clb.11_GM000714) encoding acetyl-CoA C-acetyltransferase, *INO1* (Clb.11_GM004026) encoding myo-inositol-1-phosphate synthase, and Clb.11_GM002939 encoding a GNAT family N-acetyltransferase related to biofilm synthesis were continuously upregulated as the concentration of Cr(VI) increased (Supplementary Table S6). Acetyl-CoA C-acetyltransferase is involved in fatty acid metabolism (Supplementary Table S6), which is related to biofilm synthesis (Pedrido et al., 2013). Myo-inositol-1-phosphate synthase is vital for the biogenesis of phosphatidylinositol, which is one of the materials involved in biofilm synthesis (Bachhawat and Mande, 1999). In addition, the morphology of Clb-11 cells did not continuously change with increasing Cr(VI) concentration. Therefore, the upregulated genes related to biofilm synthesis under 0.2 mM Cr(VI) were analyzed. The results showed that the genes *dhaM* (Clb.11_GM002533), *dhal* (Clb.11_GM002534), and *dhak* (Clb.11_GM002535), encoding phosphoenolpyruvate-glycerone phosphotransferase subunits DhaM, DhaL, and DhaK, respectively, and *bccA* (Clb.11_GM002750), encoding acetyl-CoA/propionyl-CoA carboxylase, were upregulated in the presence of 0.2 mM Cr(VI) (Supplementary Table S10). Phosphoenolpyruvate is related to biofilm synthesis (Houot et al., 2010). However, the genes that control cell morphology were not found in this study.

### 3.7. Changes in amino acid metabolism and other metabolic processes in Clb-11 cells in the presence of Cr(VI)

As the Cr(VI) concentration increased, Clb-11 cell growth was inhibited, and the higher the Cr(VI) concentration was, the stronger the inhibition (Figure 2D). Therefore, Cr(VI) must have a strong effect on cell metabolism. We found that the gene Clb.11_GM002939 related to 2-oxocarboxylic acid metabolism, arginine biosynthesis and biosynthesis of amino acids in Clb-11 was continuously upregulated as the concentration of Cr(VI) increased (Supplementary Table S6). However, the genes *gltD* (Clb.11_GM002736), encoding the glutamate synthase (NADPH) small chain, *glyA* (Clb.11_GM001215), encoding glycine hydroxymethyl transferase, *gpmA* (Clb.11_GM003460), encoding 2,3-bisphosphoglycerate-dependent phosphoglycerate mutase, and *pyk* (Clb.11_GM002197), encoding pyruvate kinase, were continuously downregulated as the concentration of Cr(VI) increased (Supplementary Table S7). The changes in cellular amino acid metabolism in Clb-11 cells may be related to enhanced secretion of Cr(VI) reductase and transporter proteins.

In addition, other metabolic changes occurred in Clb-11 cells in the presence of Cr(VI). The gene *pncB* (Clb.11_GM000820), encoding nicotinate phosphoribosyl transferase related to nicotinate and nicotinamide metabolism and biosynthesis of cofactors, was continuously upregulated as the concentration of Cr(VI) increased (Supplementary Table S6). Nicotinate phosphoribosyl transferase is the rate-limiting enzyme of the NAD(H) synthesis system and can catalyze the conversion of niacin (NA) to nicotinic acid mononucleotide (NAMN), which is an intermediate of the NAD^+^ synthesis pathway (Ma et al., 2013). The upregulation of *pncB* can increase the NAD(H) pool and enhance the redox level of microbial cells to reduce Cr(VI) (Jiang et al., 2022). However, the toxic effects of Cr(VI) on cellular metabolism were more pronounced. We found that the genes *ackA* (Clb.11_GM001153) encoding acetate kinase, *pta* (Clb.11_GM001154) encoding phosphate acetyltransferase, *aceE* (Clb.11_GM001681) encoding pyruvate dehydrogenase E1 component, *pgi* (Clb.11_GM002670) encoding glucose-6-phosphate isomerase, *cbhA* (Clb.11_GM002037) encoding cellulose 1,4-beta-cellobiosidase, *galU* (Clb.11_GM003247) encoding UTP--glucose-1-phosphate uridylyl transferase, *gph* (Clb.11_GM002540) encoding phosphoglycolate phosphatase, *nirB* (Clb.11_GM000112) encoding nitrite reductase (NADH) large subunit, *nirK* (Clb.11_GM002673) encoding nitrite reductase (NO-forming), *folD* (Clb.11_GM001216) encoding (NADP+)/methenyltetrahydrofolate cyclohydrolase, *purH* (Clb.11_GM001195) encoding phosphoribosylaminoimidazolecarboxamide formyltransferase/IMP cyclohydrolase, *ugd* (Clb.11_GM000269) encoding UDPglucose 6-dehydrogenase, *sacB* (Clb.11_GM001545) encoding levansucrase, *xfp* (Clb.11_GM001155) encoding xylulose-5-phosphate/fructose-6-phosphate phosphoketolase, and *feoB* and *feoA* encoding ferrous iron transport protein B and A, respectively, were continuously downregulated as the concentration of Cr(VI) increased (Supplementary Table S7). The genes *ackA* (Kuit et al., 2012), *pta* (Castaño-Cerezo et al., 2009), *pgi* (Charusanti et al., 2010), *cbhA* (Meinke et al., 1994), *galU* (Daran et al., 1995), *gph* (Lyngstadaas et al., 1999), *ugd* (Mouslim and Groisman, 2003), *xfp* (Meile et al., 2001), and *sacB* (Meng and Fütterer, 2003) are associated with cell energy production. The gene *aceE* is related to the rate of cell growth (Chen et al., 2020). The genes *nirB* (Macdonald and Cole, 1985), *nirK* (Adhikari and Rahman, 2017), *nirC* (Peakman et al., 1990), *feoB*, *feoA* (Cartron et al., 2006), and *fold* (D’Ari and Rabinowitz, 1991) are related to electron transfer in cells. The downregulation of these genes may be one of the reasons for the slow growth and progressive decrease in the OD_600 nm_ of Clb-11 with increasing Cr(VI) concentrations.

**Figure 6:**
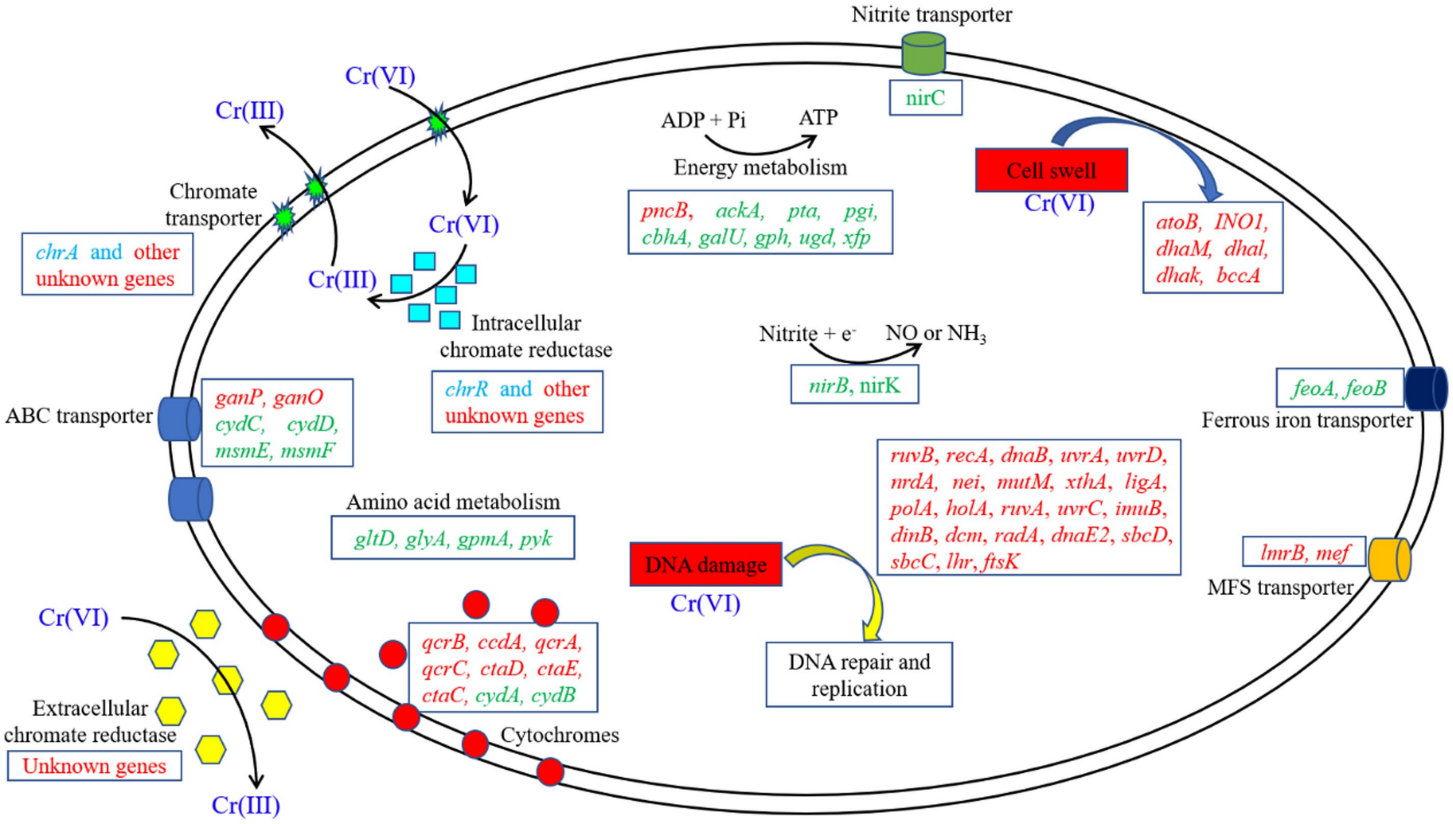
Schematic model of the metabolic adaptation of Clb-11 to different Cr(VI) concentrations. The upregulated (red) and downregulated (green) genes in the presence of Cr(VI) are highlighted. The transcription levels of some genes (blue) were extremely low.

However, we found that most of the genes that responded to Cr(VI) stress in Clb-11 cells were not annotated, and the regulatory role of these genes might be critical for the resistance of Clb-11 cells to Cr(VI) (Supplementary Tables S6, S7). Figure 6 shows a schematic representation of the cellular mechanisms activated in the Cr(VI)-adapted state of Clb-11, and a summary of the differentially transcribed genes is presented.

### 3.8. Electricity production of Clb-11 in MFCs

The electricity generation performance of Clb-11 was investigated *via* the MFCs system, and the results are shown in Supplementary Figure S10. When the cells were fed CMC, glucose and acetate, the maximum output voltage was 62.23 ± 2.33 mV, 55.56 ± 1.53 mV and 54.92 ± 1.82 mV, respectively (Supplementary Figure S10A), and the maximum output power density was 12.17 ± 2.74 mW·m^−2^, 11.02 ± 0.73 mW·m^−2^ and 12.20 ± 1.80 mW·m^−2^, respectively (Supplementary Figures S10B–D). Strain Lsc-8, in the same genus as Clb-11, was reported to produce a maximum output power density of 9.58 mW·m^−2^ with CMC as the carbon source (Cao et al., 2020). It was reported that *C. fimi* NBRC 15513 with 0.4 mM anthraquinone-2,6-disulfonate (AQDS) and CMC could produce a maximum output power density of 38.7 mW·m^−2^ (Takeuchi et al., 2017). The power density from *Cellulomonas* was much lower than that from *G. sulfurreducens* (867 mW·m^−2^) (Jiang et al., 2019). However, *G. sulfurreducens* cannot use cellulose to produce electricity (Cao et al., 2023). The planktonic cells of *Cellulomonas* in the MFCs anode chamber were the major contributor to electricity generation, which may be the main reason for the lower electricity production of *Cellulomonas* (Cao et al., 2020). In addition, *Cellulomonas* is a gram-positive bacterium that generally generates less electricity than gram-negative bacteria. The same results were obtained for other gram-positive bacteria, e.g., *Bacillus* (Nimje et al., 2010) and *Lysinibacillus* (Nandy et al., 2013). To our knowledge, this is the first report of RNA-Seq-based transcriptional profiling of an exoelectrogen exposed to different Cr(VI) concentrations, and the findings could be useful for improving exoelectrogen characteristics or culture conditions for the application of this organism in waste treatment and MFCs.

## 4. Conclusion

In this work, a facultative exoelectrogen, named Clb-11, was successfully isolated from a polluted river. Clb-11 was identified as *Cellulomonas* sp. based on whole-genome sequencing and as a relative of TMV7-A2 based on 16S rRNA gene sequencing. The whole-genome sequencing results showed that the estimated genome size was 4,486,195 bp, and 4,052 protein-coding genes were predicted. Surprisingly, Clb-11 could utilize CMC to produce electricity in MFCs and secrete extracellular chromate reductase or extracellular electron mediator to reduce Cr(VI). However, the results indicated that the Clb-11 cells swelled significantly in the presence of Cr(VI). The transcription level of Clb-11 cells varied with different Cr(VI) concentrations in LB medium. As the concentration of Cr(VI) increased, 99 genes were continuously upregulated, and 78 genes were continuously downregulated. The 99 upregulated genes were involved in nucleotide excision repair, DNA replication, homologous recombination, base excision repair, and biosynthesis of secondary metabolites. The 78 downregulated genes were involved in carbon metabolism, pyruvate metabolism, glycolysis/gluconeogenesis, methane metabolism, amino sugar and nucleotide sugar metabolism, biosynthesis of amino acids, microbial metabolism in diverse environments, biosynthesis of cofactors, ABC transporter activity, and biosynthesis of secondary metabolites. These results suggest the prominent role of these genes in Cr(VI) resistance. In addition, we found that several genes play important roles in cellular resistance to Cr(VI).

However, the known genes encoding chromate transporters and hexavalent chromium reductase were not upregulated in this study. Therefore, there may be undiscovered chromate transporter- and hexavalent chromium reductase-coding genes among the unannotated genes of Clb-11. Nevertheless, further research is needed to confirm this hypothesis.

## Data availability statement

The datasets generated for this study can be found in the GenBank repository (https://www.ncbi.nm.nih.gov/nuccore/CP110680.1/) with accession number CP110680.

## Author contributions

CL and HS wrote the main manuscript text and processed some data of the manuscript. ML, MZ, YZ, and YN processed some data and revised of the manuscript. MH, YW, TL, FC, MW, JL, and EL edited the manuscript. All authors contributed to the article and reviewed the manuscript.

## Funding

This work was financially supported by projects from the Key Scientific Research Project of Henan Higher Education Institutions (Nos. 22B180008 and 22A180023), National Scientific Research Project Cultivation Fund Project of Huanghuai University (Nos. 110721331001 and XKPY-2022001), Key Science and Technology Specific Projects of Henan Province (No. 191110110600), Key Science and Technology Innovation Project of Zhumadian City (No. 17702), Key Specialized Research and Development Breakthrough of Henan Province (No. 232102320321), and Scientific and Technological Research Project Foundation of Henan Provincial Scientific and Technological Department (Nos. 202102310479 and 222102110180).

## Conflict of interest

The authors declare that the research was conducted in the absence of any commercial or financial relationships that could be construed as a potential conflict of interest.

## Publisher’s note

All claims expressed in this article are solely those of the authors and do not necessarily represent those of their affiliated organizations, or those of the publisher, the editors and the reviewers. Any product that may be evaluated in this article, or claim that may be made by its manufacturer, is not guaranteed or endorsed by the publisher.
