# Supplementary material for: Physiological and transcriptional studies reveal Cr(VI) reduction mechanisms in the exoelectrogen Cellulomonas fimi Clb-11
Source: Front Microbiol. 2023 May 26;14:1161303. doi: 10.3389/fmicb.2023.1161303 (PMC10251745; doi:10.3389/fmicb.2023.1161303)
Supplement: Supplementary file 1 [file Data_Sheet_1.pdf]

Supplementary

Physiological and Transcriptional Studies Reveal Cr(VI) Reduction Mechanisms in the Exoelectrogen *Cellulomonas fimi* Clb-11

Lianbin Cao, Mingguo Lu, Mengrui Zhao, Yifan Zhang, Yiping Nong, Mengxue Hu, Ya Wang, Tongbiao Li, Fujia Chen, Mingcheng Wang, Junhe Liu, Enzhong Li* and Hongmei Sun*

School of Biological and Food Engineering, Huanghuai University, No. 76 Kaiyuan Road, Zhumadian, Henan Province 463000, PR China

The complete genome sequence of *Cellulomonas fimi* Clb-11 has been deposited in the NCBI database under accession number CP110680.

* Corresponding author.

Current address: School of Biological and Food Engineering, Huanghuai University, No. 76 Kaiyuan Road, Zhumadian, Henan Province 463000, PR China. E-mail address: shm58@163.com

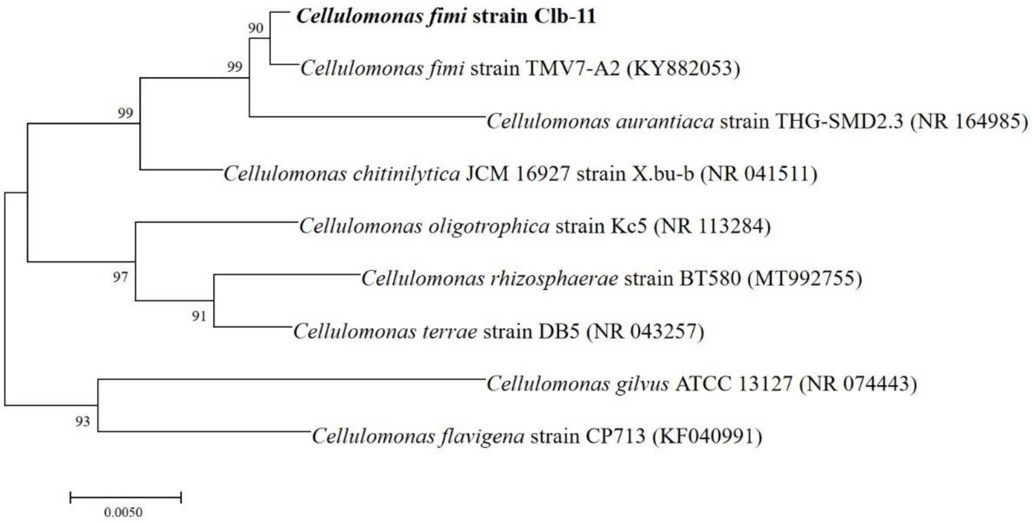

Fig. S1 Phylogenetic tree of *C. fimi* strain Clb-11 with closely related species based on 16S rDNA gene sequences. Numbers at each node present the percentages of occurrence of the branching order in 1,000 bootstrapped trees for values greater than 50%. The scale bar represents 0.5% divergence.

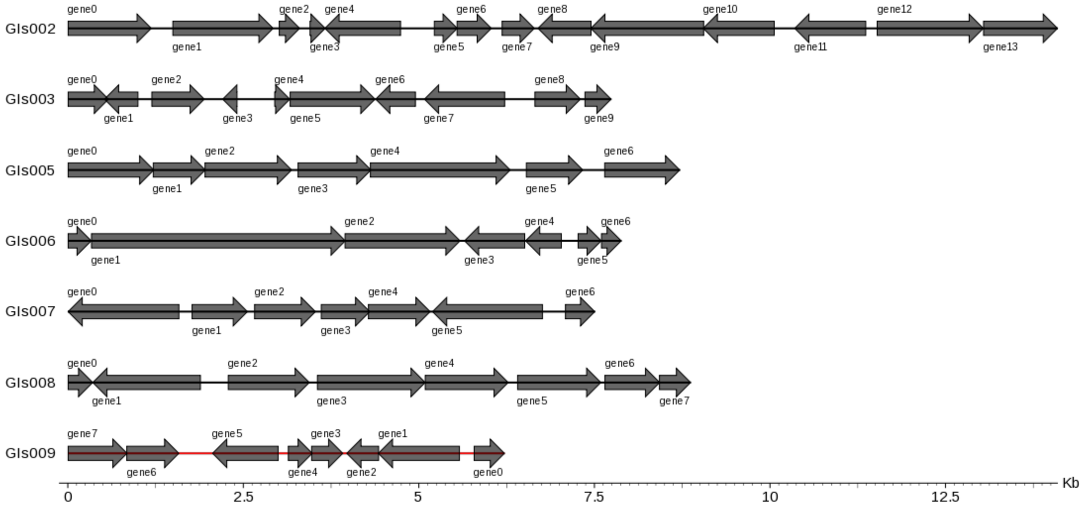

Fig. S2 Genomics island (GI) gene distribution statistical maps of *C. fimi* strain Clb-11.

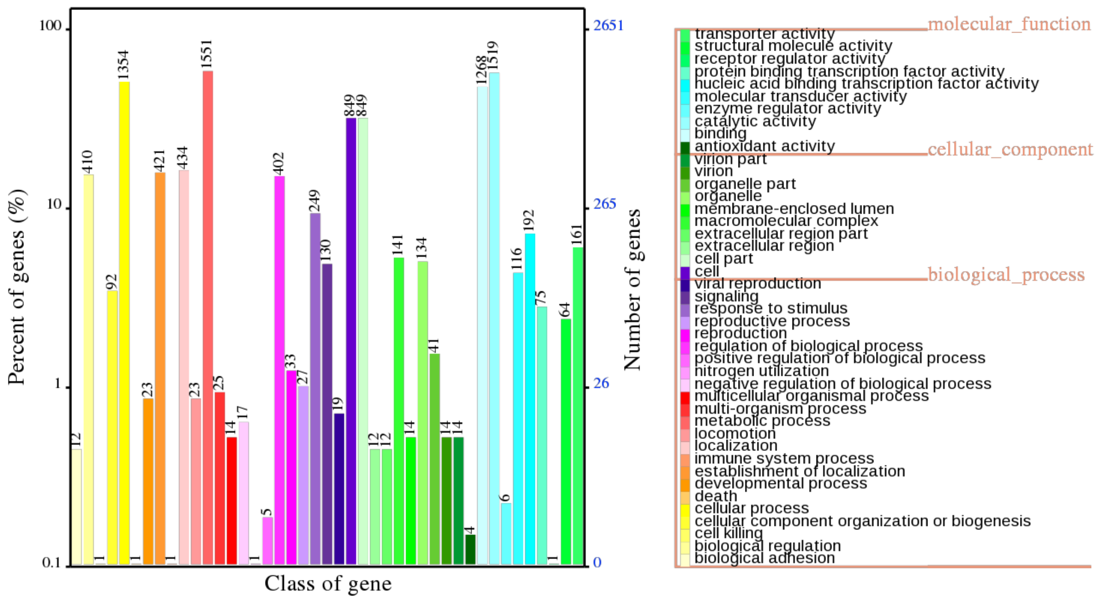

Fig. S3 Gene function annotation of *C. fimi* strain Clb-11 by Gene Ontology (GO).

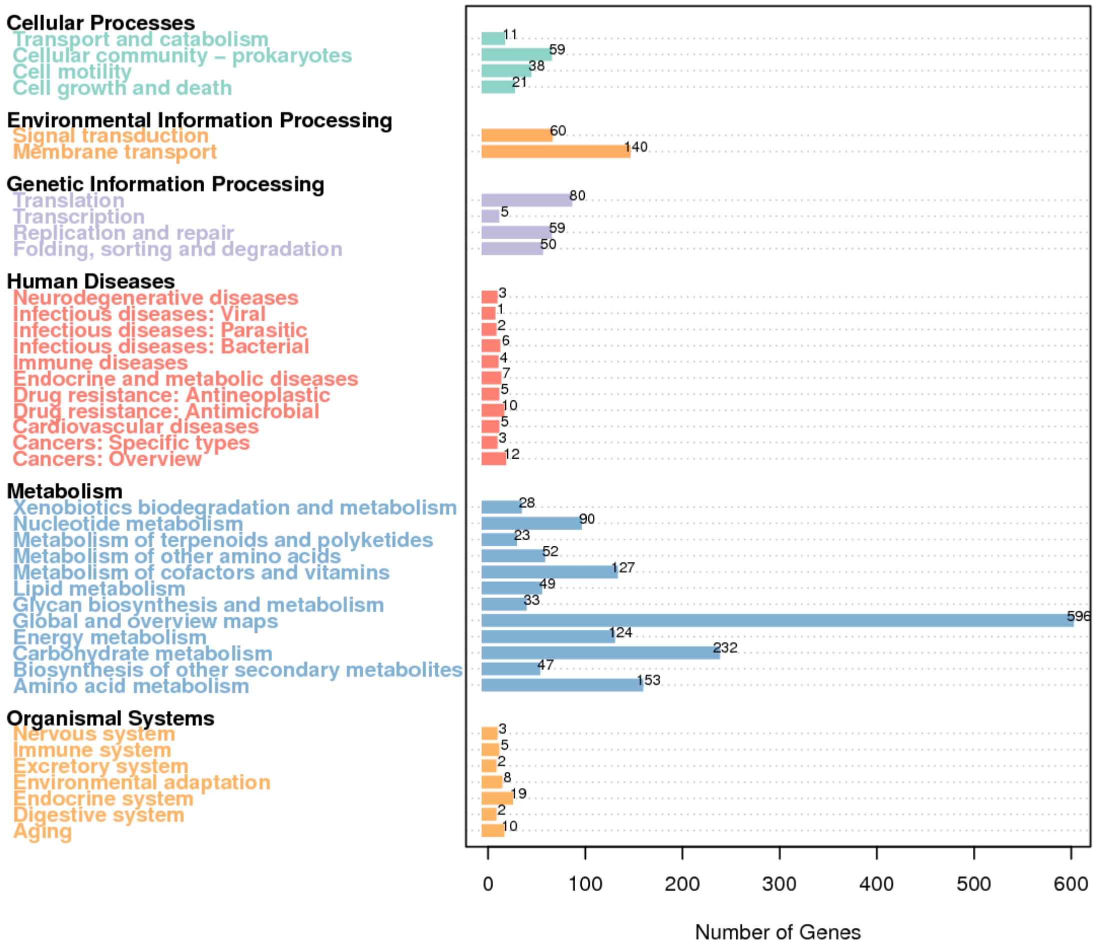

Fig. S4 Gene function annotation of *C. fimi* strain Clb-11 in eight biological pathway classifications by Kyoto Encyclopedia of Genes and Genomes (KEGG).

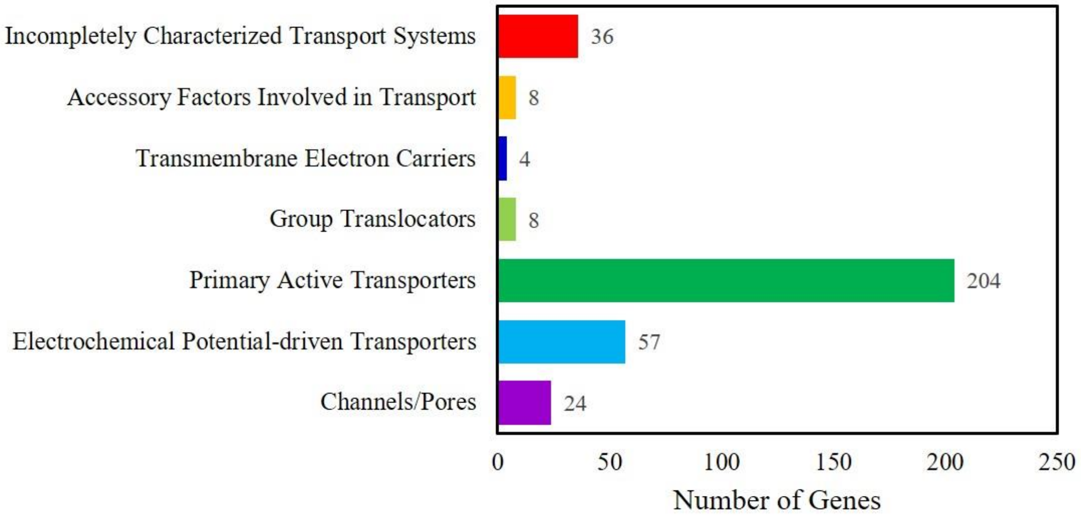

Fig. S5 Gene function annotation of *C. fimi* strain Clb-11 by the Transporter Classification Database (TCDB).

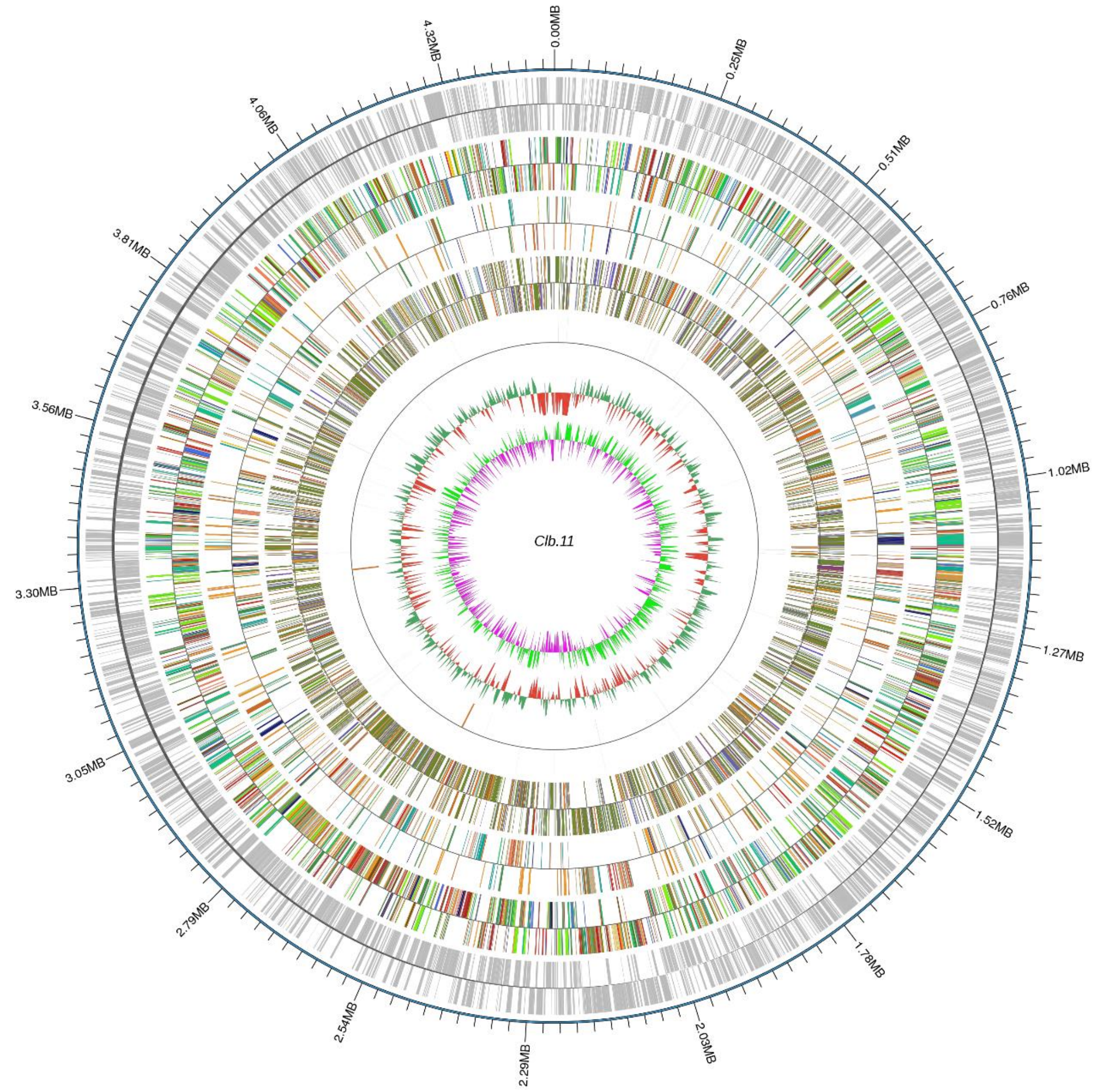

Fig. S6 The whole genome complete map of *C. fimi* strain Clb-11 with 0 gaps. The outermost circle shows the position coordinates of the genome sequence. From the outside to the inside, the circles show the contents of coding genes, gene function annotation results (include annotation results of COG (KOG), KEGG and GO databases), ncRNA and genome GC content.

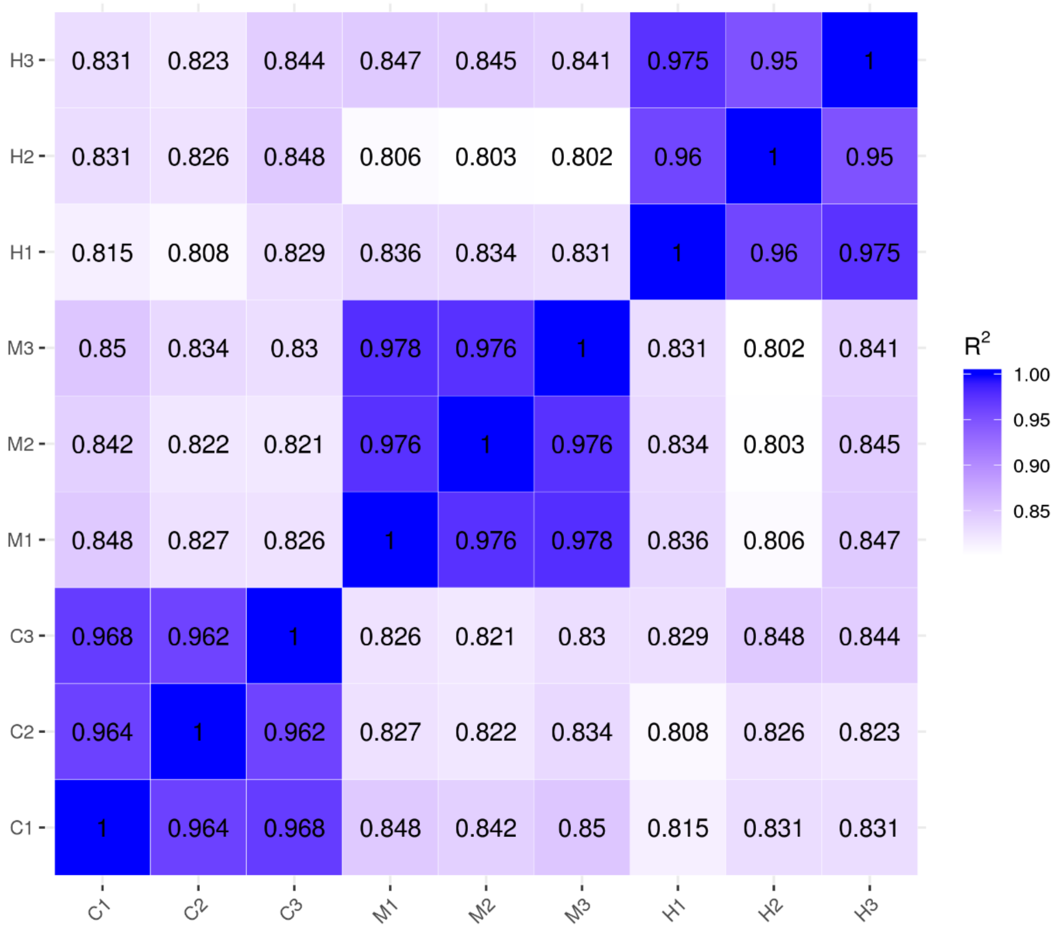

Fig. S7 Pearson correlation among *C. fimi* strain Clb-11 cell samples, *C. fimi* strain Clb-11 with 0 mM Cr(VI) (C1, C2 and C3, as three replications), 0.2 mM Cr(VI) (M1, M2 and M3, as three replications) and 0.5 mM Cr(VI) (H1, H2 and H3, as three replications).

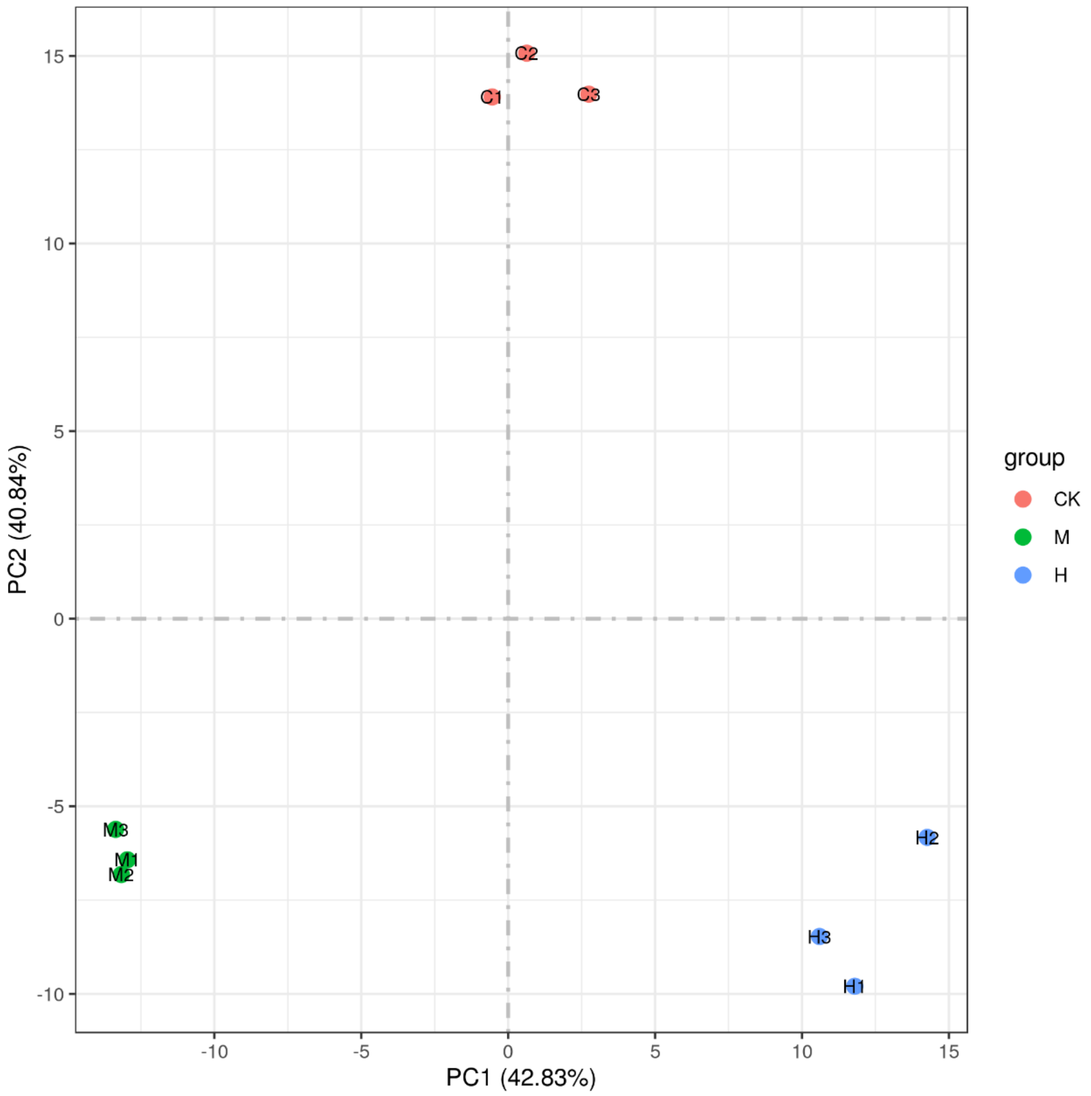

Fig. S8 Principal component analysis (PCA) of *C. fimi* strain Clb-11 cell samples, *C. fimi* strain Clb-11 with 0 mM Cr(VI) (C1, C2 and C3, as three replications), 0.2 mM Cr(VI) (M1, M2 and M3, as three replications) and 0.5 mM Cr(VI) (H1, H2 and H3, as three replications).

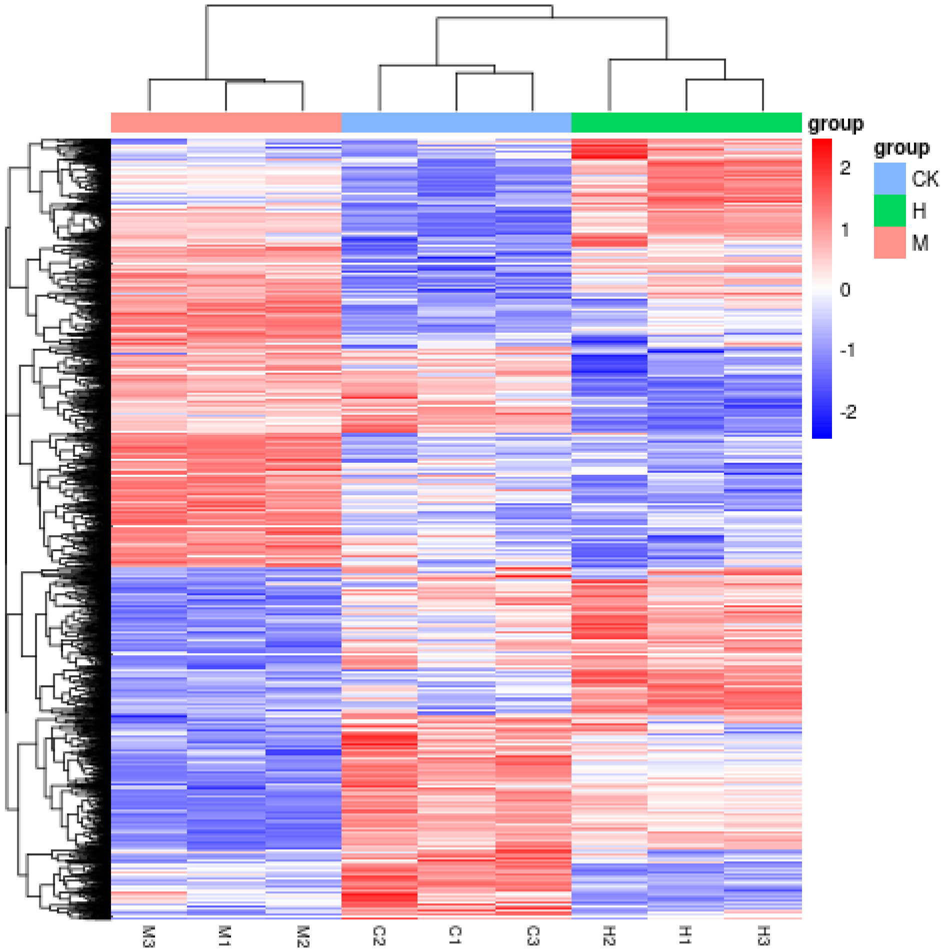

Fig. S9 The differential gene cluster heatmap of *C. fimi* strain Clb-11 cells with 0 mM Cr(VI) (C1, C2 and C3, as three replications), 0.2 mM Cr(VI) (M1, M2 and M3, as three replications), and 0.5 mM Cr(VI) (H1, H2 and H3, as three replications).

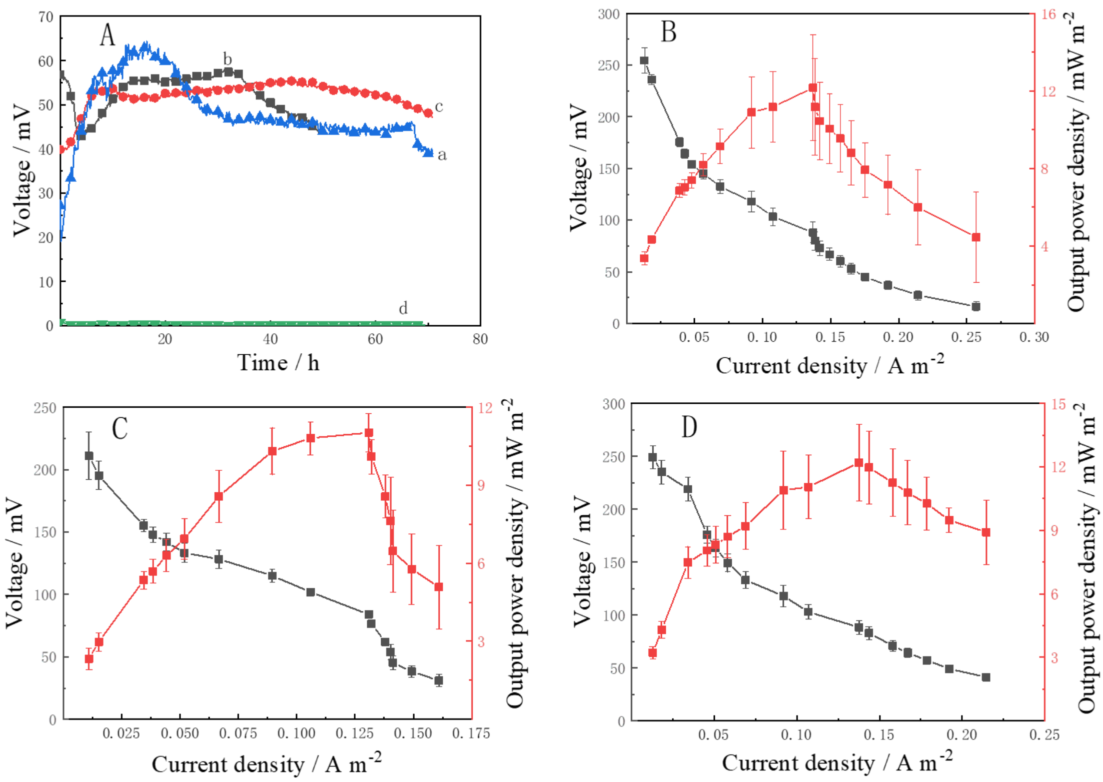

Fig. S10 Electricity production performance of strain Clb-11 in MFCs with different carbon sources. (A) Output voltage of the MFCs with CMC (curve a), glucose (curve b), and acetate (curve c) as the carbon sources and without carbon sources (curve d). (B), (C), and (D) Output power density and output voltage as a function of current density in the MFCs for Clb-11 with CMC, glucose, and acetate as the carbon sources, respectively.

Table S1 Sequencing analysis of *C. fimi* strain Clb-11

Strain	Number of Reads	Number of Bases (bp)	Mean Read Length(bp)	N_{50} Read Length(bp)	Mean Read quality
C. fimi strain Clb-11	106,980.0	1,212,413,621.0	11,333.1	12,302.0	10.9

Note: The Nanopore sequencing data were converted from fast5 format to fastq format using Guppy software basecalling, and the samples were split according to barcode using qcat software. The data split into each sample were quality controlled using NanoPlot software with a threshold of Q>7 to obtain valid data.

Table S2 Genome assembly results of *C. fimi* strain Clb-11

Strain	Type	genome size (bp)	GC%
C. fimi strain Clb-11	Chromosome	4,486,195	74.41

Table S3 Coding gene prediction results of *C. fimi* strain Clb-11

Strain	Gene number	Gene total length (bp)	Gene average length	Gene length/Genome(%)
C. fimi strain Clb-11	4052	4,088,556	1009	91.14

Table S4 Prediction of secreted protein results

Strain	SignalP protein	TMHMM protein	Secreted protein
C. fimi strain Clb-11	195	1051	102

Table S5 Cr(VI) reduction ratio of *C. fimi* strain Clb-11 in different time periods

Cr(VI) concentration (mM)	Reduction ratio of Cr(VI) ($mM \cdot h^{-1}$)							
	0 h to 24 h	24 h to 48 h	48 h to 72 h	72 h to 96 h	96 h to 120 h	120 h to 144 h	144 h to 168 h	168 h to 192 h
0.1	0.00060	0.00087	0.0013	0.0013	0.00023	--	--	--
0.2	0.0013	0.0020	0.0020	0.0026	0.00033	--	--	--
0.3	0.0035	0.0013	0.0013	0.0061	0.00025	--	--	--
0.4	0.0018	0.0028	0.0035	0.0084	0.00018	--	--	--
0.5	0.0039	0.0025	0.0025	0.011	0	0.00082	--	--
0.6	0.0036	0.0033	0.0033	0.010	0.00082	0.00071	0	0.00060
0.7	0.0040	0.0046	0.0031	0.011	0	0.00057	0.00053	0

Table S6 Annotation of 99 upregulated genes in *C. fimi* strain Clb-11 by KEGG orthology (KO)

ORF	Gene name	KO	Gene product
**Two-component system and Valine, leucine and isoleucine degradation**			
Clb.11_GM000714	*atoB*	K00626	acetyl-CoA C-acetyltransferase
**ABC transporters**			
Clb.11_GM001640	*ganP*	K15771	arabinogalactan oligomer/maltooligosaccharide transport system permease protein
Clb.11_GM001639	*ganO*	K15770	arabinogalactan oligomer/maltooligosaccharide transport system substrate-binding protein
**2-Oxocarboxylic acid metabolism, Arginine biosynthesis and Biosynthesis of amino acids**			
Clb.11_GM002939			GNAT family N-acetyltransferase
**Base excision repair**			
Clb.11_GM002457	*nei*	K05522	endonuclease VIII
Clb.11_GM002494	*mutM*	K10563	formamidopyrimidine-DNA glycosylase
Clb.11_GM000352/Clb.11_GM001218	*xthA*	K01142	exodeoxyribonuclease III
Clb.11_GM002940	*ligA*	K01972	NAD-dependent DNA ligase LigA
Clb.11_GM002149	*polA*	K02335	DNA polymerase I
**Mismatch repair**			
Clb.11_GM002940	*ligA*	K01972	NAD-dependent DNA ligase LigA
Clb.11_GM001579	*holA*	K02340	DNA polymerase III subunit delta
**Homologous recombination**			
Clb.11_GM001926	*ruvB*	K03551	holliday junction DNA helicase RuvB
Clb.11_GM001579	*holA*	K02340	DNA polymerase III subunit delta
Clb.11_GM002149	*polA*	K02335	DNA polymerase I
Clb.11_GM001925	*ruvA*	K03550	holliday junction DNA helicase RuvA

Clb.11_GM002452	*recA*	K03553	recombinase RecA
**DNA replication**			
Clb.11_GM002940	*ligA*	K01972	NAD-dependent DNA ligase LigA
Clb.11_GM001579	*holA*	K02340	DNA polymerase III subunit delta
Clb.11_GM002149	*polA*	K02335	DNA polymerase I
Clb.11_GM004012	*dnaB*	K02314	replicative DNA helicase
**Glyoxylate and dicarboxylate metabolism and Pyruvate metabolism**			
Clb.11_GM000714	*atoB*	K00626	acetyl-CoA C-acetyltransferase
**Nucleotide metabolism**			
Clb.11_GM000387	*nrdA*	K00525	ribonucleoside-diphosphate reductase alpha chain
**Fatty acid metabolism, Terpenoid backbone biosynthesis and Microbial metabolism in diverse environments**			
Clb.11_GM000714	*atoB*	K00626	acetyl-CoA C-acetyltransferase
**Nucleotide excision repair**			
Clb.11_GM002128	*uvrC*	K03703	excinuclease ABC subunit C
Clb.11_GM002940	*ligA*	K01972	NAD-dependent DNA ligase LigA
Clb.11_GM002131	*uvrA*	K03701	excinuclease ABC subunit A
Clb.11_GM002149	*polA*	K02335	DNA polymerase I
**Carbon metabolism, Butanoate metabolism and Fatty acid degradation**			
Clb.11_GM000714	*atoB*	K00626	acetyl-CoA C-acetyltransferase
**Pyrimidine metabolism**			
Clb.11_GM000387	*nrdA*	K00525	ribonucleoside-diphosphate reductase alpha chain
**Streptomycin biosynthesis**			
Clb.11_GM004026	*INO1*	K01858	myo-inositol-1-phosphate synthase
**Purine metabolism**			
Clb.11_GM000387	*nrdA*	K00525	ribonucleoside-diphosphate reductase alpha chain
**Nicotinate and nicotinamide metabolism and Biosynthesis of cofactors**			
Clb.11_GM000820	*pncB*	K0076	nicotinate phosphoribosyl

		3	transferase
**Sulfur relay system**			
Clb.11_GM003716			ThiF family adenylyltransferase
**Biosynthesis of secondary metabolites**			
Clb.11_GM000714	*atoB*	K00626	acetyl-CoA C-acetyltransferase
Clb.11_GM002939			GNAT family N-acetyltransferase
Clb.11_GM004026	*INO1*	K01858	myo-inositol-1-phosphate synthase
**Others**			
Clb.11_GM000211	*phnB*	K04750	VOC family protein
Clb.11_GM002451	*recX*	K03565	regulatory protein RecX
Clb.11_GM002278	*imuB*	K14161	Y-family DNA polymerase
Clb.11_GM002813	*dinB*	K02346	DNA polymerase IV
Clb.11_GM000067	*dcm*	K00558	DNA (cytosine-5)-methyltransferase 1
Clb.11_GM000379	*radA*	K04485	DNA repair protein RadA/Sms
Clb.11_GM002277	*dnaE2*	K14162	error-prone DNA polymerase
Clb.11_GM002431	*lexA*	K01356	transcriptional repressor LexA
Clb.11_GM003261	*lmrB*	K18926	MFS transporter, DHA2 family, lincomycin resistance protein
Clb.11_GM001221	*ABC.CD.P*	K02004	putative ABC transport system permease protein
Clb.11_GM001447	*TC.DME*	K03298	drug/metabolite transporter, DME family
Clb.11_GM001222	*ABC.CD.A*	K02003	putative ABC transport system ATP-binding protein
Clb.11_GM000385	*nrdH*	K06191	glutaredoxin-like protein NrdH
Clb.11_GM003910	*mef*	K08217	MFS transporter, DHA3 family, macrolide efflux protein
Clb.11_GM000386	*nrdI*	K03647	protein involved in ribonucleotide reduction

Clb.11_GM003172	*bglB*	K05350	beta-glucosidase
Clb.11_GM003174	*padR*	K10947	PadR family transcriptional regulator, regulatory protein PadR
Clb.11_GM001246	*whiB7*	K18958	WhiB family transcriptional regulator, redox-sensing transcriptional regulator
Clb.11_GM000184	*sbcD, Mre11*	K03547	DNA repair protein SbcD/Mre11
Clb.11_GM000185	*sbcC, rad50*	K03546	DNA repair protein SbcC/Rad50
Clb.11_GM001163	*yaeJ*	K15034	ribosome-associated protein
Clb.11_GM002456	*lhr*	K03724	ATP-dependent helicase Lhr and Lhr-like helicase
Clb.11_GM001380	*ftsK, spoIIIE*	K03466	DNA segregation ATPase FtsK/SpoIIIE, S-DNA-T family
Clb.11_GM000107	*htpG*	K04079	molecular chaperone HtpG
Clb.11_GM001182	*pcrA*	K03657	DNA helicase PcrA
Clb.11_GM000785	*troR*	K03709	DtxR family transcriptional regulator, Mn-dependent transcriptional regulator
Clb.11_GM001139	*groES*	K04078	chaperonin GroES
Clb.11_GM002416			SAV_6107 family HEPN domain-containing protein
Clb.11_GM002279			hypothetical protein
Clb.11_GM001938			Rv2578c family radical SAM protein
Clb.11_GM001223			efflux RND transporter periplasmic adaptor subunit
Clb.11_GM000356			MBL fold metallo-hydrolase
Clb.11_GM001165			ABC transporter ATP-binding protein
Clb.11_GM003992			hypothetical protein
Clb.11_GM003947			dienelactone hydrolase family protein
Clb.11_GM004025			helix-turn-helix transcriptional regulator
Clb.11_GM004005			hypothetical protein

Clb.11_GM001164	ABC transporter ATP-binding protein
Clb.11_GM001239	ATP-dependent DNA helicase
Clb.11_GM004013	MATE family efflux transporter
Clb.11_GM003325	LLM class flavin-dependent oxidoreductase
Clb.11_GM000897	HAD-IB family hydrolase
Clb.11_GM002783	hypothetical protein
Clb.11_GM003262	hypothetical protein
Clb.11_GM000515	hypothetical protein
Clb.11_GM001903	DUF4132 domain-containing protein
Clb.11_GM003213	DUF6518 family protein
Clb.11_GM000312	HNH endonuclease family protein
Clb.11_GM002656	DEAD/DEAH box helicase
Clb.11_GM004004	hypothetical protein
Clb.11_GM003732	GNAT family N-acetyltransferase
Clb.11_GM000967	class I SAM-dependent methyltransferase
Clb.11_GM003918	winged helix DNA-binding domain-containing protein
Clb.11_GM000856	Uncharacterized protein
Clb.11_GM000992	YceI family protein
Clb.11_GM000378	hypothetical protein
Clb.11_GM004003	hypothetical protein
Clb.11_GM002628	hypothetical protein
Clb.11_GM002722	patatin-like phospholipase family protein
Clb.11_GM000715	TetR/AcrR family transcriptional regulator
Clb.11_GM000315	hemolysin family protein
Clb.11_GM000056	hypothetical protein
Clb.11_GM000025	rhomboid family intramembrane serine protease
Clb.11_GM001593	DUF4870 domain-containing protein
Clb.11_GM000316	hemolysin family protein
Clb.11_GM000829	HNH endonuclease
Clb.11_GM000310	rhomboid family intramembrane serine

	protease
Clb.11_GM000350	DUF4190 domain-containing protein
Clb.11_GM000827	hypothetical protein
Clb.11_GM002723	restriction endonuclease
Clb.11_GM003847	hypothetical protein
Clb.11_GM001188	OmpA family protein
Clb.11_GM004002	hypothetical protein
Clb.11_GM003911	GyrI-like domain-containing protein
Clb.11_GM001904	AAA family ATPase
Clb.11_GM003968	hypothetical protein

Table S7 Annotation of 78 downregulated genes in *C. fimi* strain Clb-11 by KEGG orthology (KO)

ORF	Gene name	KO	Gene product
**Two-component system**			
Clb.11_GM002037	*cbhA*	K19668	cellulose 1,4-beta-cellobiosidase
Clb.11_GM001485	*cydA*	K00425	cytochrome bd ubiquinol oxidase subunit I
**Propanoate metabolism**			
Clb.11_GM001153	*ackA*	K00925	acetate kinase
Clb.11_GM001154	*pta*	K13788	phosphate acetyltransferase
**ABC transporters**			
Clb.11_GM001482	*cydC*	K16012	ATP-binding cassette, subfamily C, bacterial CydC
Clb.11_GM001483	*cydD*	K16013	ATP-binding cassette, subfamily C, bacterial CydD
Clb.11_GM002904	*msmE*	K10117	raffinose/stachyose/melibiose transport system substrate-binding protein
Clb.11_GM002903	*msmF*	K10118	raffinose/stachyose/melibiose transport system permease protein
**Oxidative phosphorylation**			
Clb.11_GM001485	*cydA*	K00425	cytochrome bd ubiquinol oxidase subunit I
Clb.11_GM001484	*cydB*	K00426	cytochrome bd ubiquinol oxidase subunit II
**Pyruvate metabolism**			
Clb.11_GM001153	*ackA*	K00925	acetate kinase
Clb.11_GM002197	*pyk*	K00873	pyruvate kinase
Clb.11_GM001681	*aceE*	K00163	pyruvate dehydrogenase E1 component
Clb.11_GM001154	*pta*	K13788	phosphate acetyltransferase
**Starch and sucrose metabolism**			
Clb.11_GM002670	*pgi*	K01810	glucose-6-phosphate isomerase
Clb.11_GM002037	*cbhA*	K19668	cellulose 1,4-beta-cellobiosidase
Clb.11_GM003247	*galU*	K00963	UTP--glucose-1-phosphate uridylyl transferase
**Alanine, aspartate and glutamate metabolism**			
Clb.11_GM002736	*gltD*	K00266	glutamate synthase (NADPH) small chain
**Glyoxylate and dicarboxylate metabolism**			
Clb.11_GM001215	*glyA*	K00600	glycine hydroxymethyl transferase
Clb.11_GM002540	*gph*	K01091	phosphoglycolate phosphatase
**Nitrogen metabolism**			
Clb.11_GM000112	*nirB*	K00362	nitrite reductase (NADH) large subunit
Clb.11_GM002673	*nirK*	K00368	nitrite reductase (NO-forming)
Clb.11_GM002736	*gltD*	K00266	glutamate synthase (NADPH) small chain
**Carbon metabolism**			
Clb.11_GM002670	*pgi*	K01810	glucose-6-phosphate isomerase
Clb.11_GM001215	*glyA*	K00600	glycine hydroxymethyl transferase
Clb.11_GM001153	*ackA*	K00925	acetate kinase
Clb.11_GM003460	*gpmA*	K01834	2,3-bisphosphoglycerate-dependent phosphoglycerate mutase

Clb.11_GM002197	*pyk*	K00873	pyruvate kinase
Clb.11_GM001216	*folD*	K01491	methylenetetrahydrofolate dehydrogenase (NADP+)/methenyltetrahydrofolate cyclohydrolase
Clb.11_GM001681	*aceE*	K00163	pyruvate dehydrogenase E1 component
Clb.11_GM001154	*pta*	K13788	phosphate acetyltransferase
**Glycine, serine and threonine metabolism**			
Clb.11_GM001215	*glyA*	K00600	glycine hydroxymethyl transferase
Clb.11_GM003460	*gpmA*	K01834	2,3-bisphosphoglycerate-dependent phosphoglycerate mutase
**Glycolysis/Gluconeogenesis**			
Clb.11_GM002670	*pgi*	K01810	glucose-6-phosphate isomerase
Clb.11_GM003460	*gpmA*	K01834	2,3-bisphosphoglycerate-dependent phosphoglycerate mutase
Clb.11_GM002197	*pyk*	K00873	pyruvate kinase
Clb.11_GM001681	*aceE*	K00163	pyruvate dehydrogenase E1 component
**Citrate cycle (TCA cycle)**			
Clb.11_GM001681	*aceE*	K00163	pyruvate dehydrogenase E1 component
**Purine metabolism**			
Clb.11_GM001195	*purH*	K00602	phosphoribosylaminoimidazolecarboxamide formyltransferase/IMP cyclohydrolase
**One carbon pool by folate**			
Clb.11_GM001215	*glyA*	K00600	glycine hydroxymethyl transferase
Clb.11_GM001216	*folD*	K01491	methylenetetrahydrofolate dehydrogenase (NADP+)/methenyltetrahydrofolate cyclohydrolase
Clb.11_GM001195	*purH*	K00602	phosphoribosylaminoimidazolecarboxamide formyltransferase/IMP cyclohydrolase
**Methane metabolism**			
Clb.11_GM001215	*glyA*	K00600	glycine hydroxymethyl transferase
Clb.11_GM001153	*ackA*	K00925	acetate kinase
Clb.11_GM003460	*gpmA*	K01834	2,3-bisphosphoglycerate-dependent phosphoglycerate mutase
Clb.11_GM001154	*pta*	K13788	phosphate acetyltransferase
**Pentose and glucuronate interconversions**			
Clb.11_GM000269	*ugd*	K00012	UDPglucose 6-dehydrogenase
Clb.11_GM003247	*galU*	K00963	UTP--glucose-1-phosphate uridylyl transferase
**Galactose metabolism**			
Clb.11_GM003247	*galU*	K00963	UTP--glucose-1-phosphate uridylyl transferase
**Pentose phosphate pathway**			
Clb.11_GM002670	*pgi*	K01810	glucose-6-phosphate isomerase
Clb.11_GM001155	*xfp*	K01621	xylulose-5-phosphate/fructose-6-phosphate phosphoketolase
**Amino sugar and nucleotide sugar metabolism**			

Clb.11_GM002670	*pgi*	K01810	glucose-6-phosphate isomerase
Clb.11_GM003311	E3.2.1.14	K01183	chitinase
Clb.11_GM000269	*ugd*	K00012	UDPglucose 6-dehydrogenase
Clb.11_GM003139			AraC family transcriptional regulator
Clb.11_GM003247	*galU*	K00963	UTP--glucose-1-phosphate uridylyl transferase
**O-Antigen nucleotide sugar biosynthesis**			
Clb.11_GM000269	*ugd*	K00012	UDPglucose 6-dehydrogenase
Clb.11_GM003247	*galU*	K00963	UTP--glucose-1-phosphate uridylyl transferase
**Biosynthesis of nucleotide sugars**			
Clb.11_GM002670	*pgi*	K01810	glucose-6-phosphate isomerase
Clb.11_GM000269	*ugd*	K00012	UDPglucose 6-dehydrogenase
Clb.11_GM003247	*galU*	K00963	UTP--glucose-1-phosphate uridylyl transferase
**Biosynthesis of amino acids**			
Clb.11_GM001215	*glyA*	K00600	glycine hydroxymethyl transferase
Clb.11_GM003460	*gpmA*	K01834	2,3-bisphosphoglycerate-dependent phosphoglycerate mutase
Clb.11_GM002197	*pyk*	K00873	pyruvate kinase
Clb.11_GM002736	*gltD*	K00266	glutamate synthase (NADPH) small chain
**Porphyrin metabolism**			
Clb.11_GM001259			TIGR04053 family radical SAM/SPASM domain-containing protein
**Microbial metabolism in diverse environments**			
Clb.11_GM002670	*pgi*	K01810	glucose-6-phosphate isomerase
Clb.11_GM001215	*glyA*	K00600	glycine hydroxymethyl transferase
Clb.11_GM000112	*nirB*	K00362	nitrite reductase (NADH) large subunit
Clb.11_GM001153	*ackA*	K00925	acetate kinase
Clb.11_GM003460	*gpmA*	K01834	2,3-bisphosphoglycerate-dependent phosphoglycerate mutase
Clb.11_GM002197	*pyk*	K00873	pyruvate kinase
Clb.11_GM001216	*folD*	K01491	methylenetetrahydrofolate dehydrogenase (NADP+)/methenyltetrahydrofolate cyclohydrolase
Clb.11_GM001681	*aceE*	K00163	pyruvate dehydrogenase E1 component
Clb.11_GM002673	*nirK*	K00368	nitrite reductase (NO-forming)
Clb.11_GM001154	*pta*	K13788	phosphate acetyltransferase
Clb.11_GM001155	*xfp*	K01621	xylulose-5-phosphate/fructose-6-phosphate phosphoketolase
Clb.11_GM002736	*gltD*	K00266	glutamate synthase (NADPH) small chain
**Biosynthesis of cofactors**			
Clb.11_GM001215	*glyA*	K00600	glycine hydroxymethyl transferase
Clb.11_GM001216	*folD*	K01491	methylenetetrahydrofolate dehydrogenase (NADP+)/methenyltetrahydrofolate cyclohydrolase
Clb.11_GM001259			TIGR04053 family radical SAM/SPASM

			domain-containing protein
Clb.11_GM000269	*ugd*	K00012	UDPglucose 6-dehydrogenase
Clb.11_GM003247	*galU*	K00963	UTP--glucose-1-phosphate uridylyl transferase
**Biosynthesis of secondary metabolites**			
Clb.11_GM002670	*pgi*	K01810	glucose-6-phosphate isomerase
Clb.11_GM001215	*glyA*	K00600	glycine hydroxymethyl transferase
Clb.11_GM002540	*gph*	K01091	phosphoglycolate phosphatase
Clb.11_GM000360	*glpD*	K00111	glycerol-3-phosphate dehydrogenase
Clb.11_GM003460	*gpmA*	K01834	2,3-bisphosphoglycerate-dependent phosphoglycerate mutase
Clb.11_GM002197	*pyk*	K00873	pyruvate kinase
Clb.11_GM001681	*aceE*	K00163	pyruvate dehydrogenase E1 component
Clb.11_GM001259			TIGR04053 family radical SAM/SPASM domain-containing protein
Clb.11_GM001195	*purH*	K00602	Phosphoribosylaminoimidazole carboxamide formyl transferase/IMP cyclohydrolase
Clb.11_GM002736	*gltD*	K00266	glutamate synthase (NADPH) small chain
Clb.11_GM003247	*galU*	K00963	UTP--glucose-1-phosphate uridylyl transferase
**Others**			
Clb.11_GM001207	*sprC*	K18546	Streptogrisin C
Clb.11_GM000079	*feoB*	K04759	ferrous iron transport protein B
Clb.11_GM000080	*feoA*	K04758	ferrous iron transport protein A
Clb.11_GM003226	*rpfB*	K21688	resuscitation-promoting factor RpfB
Clb.11_GM000267	*hasA*	K00752	hyaluronan synthase
Clb.11_GM001545	*sacB*	K00692	levansucrase
Clb.11_GM000893	*nirC*	K02598	nitrite transporter
Clb.11_GM000359	*GLPF*	K02440	glycerol uptake facilitator protein
Clb.11_GM002584	*TC.GNTP*	K03299	gluconate:H+ symporter, GntP family
Clb.11_GM003931			M28 family metallopeptidase
Clb.11_GM001581			EAL domain-containing protein
Clb.11_GM001583			transcriptional regulator
Clb.11_GM000942			hypothetical protein
Clb.11_GM002642			hypothetical protein
Clb.11_GM003764			alpha/beta hydrolase
Clb.11_GM003436			alanine racemase
Clb.11_GM003437			MFS transporter
Clb.11_GM003438			TetR family transcriptional regulator
Clb.11_GM002289			SDR family oxidoreductase
Clb.11_GM000324			aminoglycoside phosphotransferase
Clb.11_GM001582			hypothetical protein
Clb.11_GM002674			hypothetical protein
Clb.11_GM002195			hypothetical protein
Clb.11_GM001487			YihY/virulence factor BrkB family protein
Clb.11_GM000808			hypothetical protein

Clb.11_GM000334	YhgE/Pip domain-containing protein
Clb.11_GM000333	hypothetical protein
Clb.11_GM001865	hypothetical protein
Clb.11_GM000268	glycosyl hydrolase
Clb.11_GM000766	peptidase
Clb.11_GM003327	phage holin family protein
Clb.11_GM002675	helix-turn-helix domain-containing protein
Clb.11_GM001304	hypothetical protein
Clb.11_GM001866	hypothetical protein
Clb.11_GM000017	hypothetical protein
Clb.11_GM000308	GPP34 family phosphoprotein
Clb.11_GM000813	hypothetical protein
Clb.11_GM000259	APC family permease
Clb.11_GM002268	LacI family DNA-binding transcriptional regulator
Clb.11_GM003925	antitoxin
Clb.11_GM002478	hypothetical protein
Clb.11_GM001885	hypothetical protein
Clb.11_GM002682	GDSL-type esterase/lipase family protein
Clb.11_GM000470	hypothetical protein
Clb.11_GM003875	PQQ-dependent sugar dehydrogenase
Clb.11_GM002545	phosphoribosyltransferase
Clb.11_GM003708	hypothetical protein
Clb.11_GM000264	aminoglycoside phosphotransferase family protein
Clb.11_GM000016	GNAT family protein
Clb.11_GM001105	hypothetical protein
Clb.11_GM001680	hypothetical protein
Clb.11_GM001357	MarR family winged helix-turn-helix transcriptional regulator

Table S8 Expression levels of known chromate reductase and transporter coding genes in Clb-11

	ORF	Gene name	Fragments Per Kilobase of exon model per Million mapped fragments (FPKM)			**Ref.**
			C	M	H	
chromate transporter	Clb.11_GM003029	*ChrA*	24.31±4.69	16.82±2.37	14.45±2.84	[1]
	----	*ChrB*	----	----	----	[2]
chromate reductase	Clb.11_GM003425	*ChrR*	306.87±25.44	207.07±17.22	254.94±19.22	[3]
	----	*YieF*	----	----	----	[4]
	----	*FerB*	----	----	----	[5]
	----	*NfsA* *NfsB*	----	----	----	[6]
	----	*MtrC*	----	----	----	[7]
	----	*OmcA*	----	----	----	
	----	*NemA*	----	----	----	[8]
	----	*AzoR*	----	----	----	[9]
	----	*Frp*	----	----	----	
	----	*YcnD*	----	----	----	
	----	*NfoR*	----	----	----	[10]
	----	*NitR*	----	----	----	[1]
	----	*NfrA*	----	----	----	[11]

Note: *C. fimi* strain Clb-11 with (C) 0 mM Cr(VI), (M) 0.2 mM Cr(VI), and (H) 0.5 mM Cr(VI) in the medium.

Table S9 Cytochrome-encoding gene transcriptional differential levels in *C. fimi* strain Clb-11

ORF	Gene name	KO	Gene product	Fragments Per Kilobase of exon model per Million mapped fragments (FPKM)		
				C	M	H
Clb.11_GM001787	*qcrB*	K03891	ubiquinol-cytochrome c reductase cytochrome b subunit	$809.05±218.04^{a}$	$424.93±38.83^{b}$	$1062.28±47.96^{c}$
Clb.11_GM001485	*cydA*	K00425	cytochrome bd ubiquinol oxidase subunit I	$618.25±132.07^{a}$	$370.89±21.30^{b}$	$28.57±9.54^{c}$
Clb.11_GM001767	*ccdA*	K06196	cytochrome c-type biogenesis protein	$666.10±179.85^{a}$	$411.86±32.57^{b}$	$915.06±34.45^{c}$
Clb.11_GM000996	*ccdA*	K06196	cytochrome c-type biogenesis protein	$128.58±22.90^{a}$	$84.12±3.99^{b}$	$95.82±3.74^{b}$
Clb.11_GM002325	*ctaA*	K02259	cytochrome c oxidase assembly protein subunit 15	$107.72±8.83^{a}$	$72.08±9.36^{b}$	$84.16±18.63^{b}$
Clb.11_GM001484	*cydB*	K00426	cytochrome bd ubiquinol oxidase subunit II	$280.72±73.00^{a}$	$225.50±12.64^{b}$	$15.45±8.47^{c}$
Clb.11_GM001788	*qcrA*	K03890	ubiquinol-cytochrome c reductase iron-sulfur subunit	$683.15±277.64^{a}$	$567.42±30.03^{a}$	$1115.50±37.26^{b}$
Clb.11_GM001789	*qcrC*	K03889	ubiquinol-cytochrome c reductase cytochrome c subunit	$604.92±164.18^{a}$	$601.22±24.98^{a}$	$1187.67±42.11^{b}$
Clb.11_GM001766	*ctaD*	K02274	cytochrome c oxidase subunit I	$576.63±185.84^{a}$	$510.68±13.25^{a}$	$877.51±43.35^{b}$
Clb.11_GM001790	*ctaE*	K02276	cytochrome c oxidase subunit III	$522.78±103.27^{a}$	$629.52±46.77^{a}$	$1104.53±51.33^{b}$
Clb.11_GM001765	*ctaC*	K02275	cytochrome c oxidase subunit II	$419.29±101.65^{a}$	$485.57±30.72^{a}$	$786.58±57.49^{b}$

Note: *C. fimi* strain Clb-11 with (C) 0 mM Cr(VI), (M) 0.2 mM Cr(VI), and (H) 0.5 mM Cr(VI) in the medium. The data in each row were analyzed for significant differences ($P<0.05$).

Table S10 Upregulated genes related to phospholipid metabolism of *C. fimi* strain Clb-11 in the presence of 0.2 mM Cr(VI)

ORF	Gene name	KO	Gene product
Clb.11_GM002533	*dhaM*	K05881	phosphoenolpyruvate---glycerone phosphotransferase subunit DhaM
Clb.11_GM002534	*dhaL*	K05879	phosphoenolpyruvate---glycerone phosphotransferase subunit DhaL
Clb.11_GM002535	*dhaK*	K05878	phosphoenolpyruvate---glycerone phosphotransferase subunit DhaK
Clb.11_GM002750	*bccA*	K11263	acetyl-CoA/propionyl-CoA carboxylase, biotin carboxylase, biotin carboxyl carrier protein

References

[1] Chai L, Ding C, Tang C, et al. Discerning three novel chromate reduce and transport genes of highly efficient Pannonibacter phragmitetus BB: From genome to gene and protein [J]. Ecotoxicology and Environmental Safety, 2018, 162:139-146.

[2] Branco R, Morais P V. Identification and Characterization of the Transcriptional Regulator ChrB in the Chromate Resistance Determinant of Ochrobactrum tritici 5bvl1 [J]. PLoS ONE, 2013, 8(11): 77987.

[3] Eswaramoorthy S, Poulain S, Hienerwadel R, et al. Crystal Structure of ChrR—A Quinone Reductase with the Capacity to Reduce Chromate [J]. PLoS ONE, 2012, 7(4):36017.

[4] Shahpiri A. Enhancement of chromate bioaccumulation by engineered Escherichia coli cells co-expressing chromate reductase (YieF) and a rice metallothionein isoform (OsMT1) [J]. Journal of Chemical Technology & Biotechnology, 2021, 96(5).

[5] Mazoch J , R Tesaík, V Sedláek, et al. Isolation and biochemical characterization of two soluble iron(III) reductases from Paracoccus denitrificans [J]. European Journal of Biochemistry, 2004, 271.

[6] Ackerley D F, Gonzalez C F, Keyhan M, et al. Mechanism of chromate reduction by the Escherichia coli protein, NfsA, and the role of different chromate reductases in minimizing oxidative stress during chromate reduction [J]. Environmental Microbiology, 2010, 6(8):851-860.

[7] Belchik S M, Kennedy D W, Dohnalkova A C, et al. Extracellular Reduction of Hexavalent Chromium by Cytochromes MtrC and OmcA of Shewanella oneidensis MR-1 [J]. Applied and Environmental Microbiology, 2011, 77(12).

[8] Robins KJ, Hooks DO, Rehm BHA, et al. Escherichia coli NemA is an efficient chromate reductase that can be biologically immobilized to provide a cell-free system for remediation of hexavalent chromium [J]. PLoS ONE. 2013.

[9] Thatoi H, Das S, Mishra J, et al.Bacterial chromate reductase, a potential enzyme for bioremediation of hexavalent chromium:a review[J].Journal of Environmental Management, 2014, 146:383-399.

[10] Han H, Song P, Cai Z, et al. Immobilizing chromate reductase NfoR on magnetic biochar reduced Cr(VI) in copper-containing wastewater [J]. Journal of cleaner production, 2022, 361.

[11] Zheng Z, Li Y, Zhang X, et al. A Bacillus subtilis strain can reduce hexavalent chromium to

trivalent and nfrA gene is involved [J]. International Biodeterioration and Biodegradation. 2015; 97: 90-96.
